# Rapid generation of ecologically relevant behavioral novelty in experimental cichlid hybrids

**DOI:** 10.1002/ece3.6471

**Published:** 2020-06-16

**Authors:** Anna F. Feller, Oliver M. Selz, Matthew D. McGee, Joana I. Meier, Salome Mwaiko, Ole Seehausen

**Affiliations:** ^1^ Division of Aquatic Ecology and Evolution Institute of Ecology and Evolution University of Bern Bern Switzerland; ^2^ Department of Fish Ecology and Evolution Centre of Ecology, Evolution and Biogeochemistry EAWAG Swiss Federal Institute of Aquatic Science and Technology Kastanienbaum Switzerland; ^3^ School of Biological Sciences Monash University Clayton Vic. Australia; ^4^ Department of Zoology University of Cambridge Cambridge UK; ^5^ St John’s College University of Cambridge Cambridge UK

**Keywords:** adaptive radiation, cichlids, hybridization, morphology, QTL mapping, sand sifting feeding behavior, transgressive segregation

## Abstract

The East African cichlid radiations are characterized by repeated and rapid diversification into many distinct species with different ecological specializations and by a history of hybridization events between nonsister species. Such hybridization might provide important fuel for adaptive radiation. Interspecific hybrids can have extreme trait values or novel trait combinations and such transgressive phenotypes may allow some hybrids to explore ecological niches neither of the parental species could tap into. Here, we investigate the potential of second‐generation (F2) hybrids between two generalist cichlid species from Lake Malawi to exploit a resource neither parental species is specialized on: feeding by sifting sand. Some of the F2 hybrids phenotypically resembled fish of species that are specialized on sand sifting. We combined experimental behavioral and morphometric approaches to test whether the F2 hybrids are transgressive in both morphology and behavior related to sand sifting. We then performed a quantitative trait loci (QTL) analysis using RADseq markers to investigate the genetic architecture of morphological and behavioral traits. We show that transgression is present in several morphological traits, that novel trait combinations occur, and we observe transgressive trait values in sand sifting behavior in some of the F2 hybrids. Moreover, we find QTLs for morphology and for sand sifting behavior, suggesting the existence of some loci with moderate to large effects. We demonstrate that hybridization has the potential to rapidly generate novel and ecologically relevant phenotypes that may be suited to a niche neither of the parental species occupies. Interspecific hybridization may thereby contribute to the rapid generation of ecological diversity in cichlid radiations.

## Graphical abstract

Interspecific hybrids can have transgressive phenotypes that may allow them to explore novel ecological niches. Using a combination of behavioral assays, morphometric analyses, and genetic mapping, we demonstrate transgressive segregation in morphology and in a novel trophic behavior in F2 hybrids between two generalist cichlid species from Lake Malawi. Such rapid generation of novel ecologically relevant phenotypes might contribute to the rapid generation of ecological diversity in cichlid fish adaptive radiations.

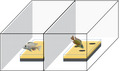

## 1 INTRODUCTION

Adaptive radiation describes the phenomenon of rapid diversification of a single lineage into an array of many species with ecologically varied adaptations (Losos, 2010; Schluter, 20002000). In the East African cichlid radiations, flocks of tens to hundreds of phenotypically diverse species have arisen in each of the major lakes in the region (Kocher, 2004; Seehausen, 2006). Phenotypic diversity in cichlids includes variation in behavior, morphology, color, and ecological specialization (Simakov et al., 2014; Greenwood, 1974; Salzburger, 2018; Seehausen, 1996).

The rate at which new species arise during adaptive radiation is often too high for new relevant mutations to emerge between successive speciation events (Hedrick, 2013). Hence, the high levels of heritable variation required in this process are likely to primarily stem from standing variation (Barrett & Schluter, 2008; Meier et al., 2017). Interspecific hybridization can rapidly generate high levels of heritable variation (Anderson & Stebbins, 1954; Bell & Travis, 2005; Hedrick, 2013; Lewontin & Birch, 1966; Seehausen, 2004). Especially hybridization between nonsister species also has the potential to reshuffle genetic variation beyond what may segregate within a population or within a group of incipient species with ongoing gene flow. Hybridization thus has the potential to provide the raw material for phenotypic novelty (Abbott et al., 2013; Stelkens, Schmid, Selz, & Seehausen, 2009), rapid adaptation (Lewontin & Birch, 1966; Stebbins, 1959), and adaptive radiation (Grant & Grant, 1992; Martin & Richards, 2019).

There is evidence for hybrid speciation in plants as well as animals (Lamichhaney et al., 2017; Mallet, 2007), and hybridization is a common feature in adaptive radiations (Marques, Meier, & Seehausen, 2019; Seehausen, 2004). In the “hybrid swarm origin” scenario (Seehausen, 2004), hybridization between distantly related lineages initiates an adaptive radiation (e.g., Barrier, Baldwin, Robichaux, & Purugganan, 1999; Hudson, Vonlanthen, & Seehausen, 2011; Joyce et al., 2011; Meier et al., 2017, 2019; Svardal et al., 2020) whereas in the “syngameon” scenario (Seehausen, 2004), occasional hybridization between nonsister member species of an adaptive radiation facilitates further diversification (e.g., Grant & Grant, 2008; Keller et al., 2013; Lamichhaney et al., 2015; McGee et al., (in press); Meier et al., 2017; Meyer, Matschiner, & Salzburger, 2017; Schliewen & Klee, 2004).

One way in which hybridization could facilitate adaptive diversification is by transgressive segregation—the occurrence of hybrid phenotypes with extreme trait values that exceed the range of trait values of both parental species combined (Rieseberg, Archer, & Wayne, 1999; Slatkin & Lande, 1994). While transgressive trait values may reduce hybrid fitness in both parental environments (Arnegard et al., 2014; Schluter, 2001), it can also allow the exploitation of a new habitat and niche that is unavailable to either parental species (Lamichhaney et al., 2017; Lexer, Welch, Raymond, & Rieseberg, 2003; Pereira, Barreto, & Burton, 2014; Selz & Seehausen, 2019). If transgressive phenotypes can be genetically stabilized and if niche differentiation then leads to reproductive isolation from the parental species, transgressive novelty can result in speciation (Mallet, 2007; Rieseberg et al., 1999). Recently, theoretical work by Kagawa & Takimoto (2017) has demonstrated that in environments with many different ecological niches, hybridization by means of transgressive segregation can facilitate the process of adaptive radiation. Alternatively, hybrid phenotypes can resemble one of the parental species, can be intermediate to both, or can combine parental traits into new trait combinations and the latter, as well as intermediate types could also allow occupation of an otherwise underutilized niche requiring intermediate or combinations of parental trait values (DeMarais, Dowling, Douglas, Minckley, & Marsh, 1992; Grant & Grant, 1996; Mallet, 2007).

Usually, transgressive segregation is due to segregation variance, sometimes due to overdominance or epistasis (Abbott et al., 2013; Rieseberg et al., 1999). Segregation variance typically results from recombination between parental species when quantitative trait loci (QTL) with antagonistic effects are present within each parental lineage and different between them, which have additive effects when recombined in the hybrid offspring (Abbott et al., 2013; Rieseberg et al., 1999; Rieseberg, Widmer, Arntz, & Burke, 2003; Seehausen, 2004). These requirements for transgressive segregation seem to be common in both plants and animals (Rieseberg et al., 2003). For example, transgressive segregation has been demonstrated for skull shape in interspecific cichlid hybrids (Albertson & Kocher, 2005). Even when individual morphological traits do not segregate outside the parental range, new combinations of traits may still result in functional or mechanical transgression (Holzman & Hulsey, 2017; Parnell, Hulsey, & Streelman, 2008). Furthermore, covariation between traits can be relaxed in hybrids, which may facilitate expansion into new areas of morphospace through the novel combination of traits (Parsons, Son, & Craig Albertson, 2011; Selz, Lucek, Young, & Seehausen, 2014). While reduced covariation in morphological traits could result in the loss of adaptation in an already adapted population on the one hand, the associated release of populations from evolutionary constraints on the other hand may promote evolvability and adaptability in new and changed environments to which a population is not adapted yet (Parsons, Son, et al., 2011).

Here, we investigate the potential of second‐generation (F2) hybrids between two generalist Lake Malawi cichlid species to exploit a novel ecological niche. The two parental species belong to two out of seven main lineages within the radiation (Malinsky et al., 2018), *Astatotilapia calliptera* and *Protomelas taeniolatus*. Previous studies of this cross have shown high levels of transgression in morphology in both first‐ (F1) and second (F2)‐generation hybrids (Selz, Lucek, et al., 2014; Stelkens et al., 2009), as well high levels of transgression in colorspace in the F1 hybrids. The latter resulted in assortative mating among F1 hybrids and parental species in mate choice experiments (Selz, Thommen, Maan, & Seehausen, 2014). Such transgression in hybrid female mating preferences favoring novel trait combinations in the hybrid males over those of the parental species was suggested to possibly facilitate the establishment of hybrid populations as incipient species, even in geographical proximity to the parental species (Selz, Thommen, et al., 2014).

The current study aimed to expand on these findings by testing whether the F2 hybrids of this cross between two generalist Malawi cichlids have the ability to exploit a new ecological niche distinct from both parents. Our specific test was motivated because some of the F2 hybrids in this cross had phenotypes that based on a qualitative impression of body shape and color, looked similar to those observed in a large clade of sand sifting species from Lake Malawi and were observed to frequently engage in sand sifting in the stock aquaria (OS personal observations). Sand sifting in search for food is an ecological specialization that has evolved multiple times independently and characterizes large clades of species in several of the large African great lake radiations of cichlid fish (Fryer & Iles, 1972; Konings, 2010).

To more thoroughly assess and characterize the occurrence of transgression and novelty in regard to sand sifting in these F2 hybrids, we took a novel approach that integrated experimental tests of transgressive performance with morphological and genetic analyses. Using a newly developed behavioral assay with two differently colored layers of sand, we assessed frequency and efficiency of sand sifting behavior in both parental species and their F2 hybrids. We used linear and geometric morphometrics to quantify variation in morphology. For trait mapping, we generated a linkage map from RAD‐seq data and performed QTL mapping. To gauge our observations in the hybrids, we also tested individuals from some specialized sand sifting species in the same setup.

We demonstrate that in addition to transgression in morphology and the appearance of novel trait combinations, some of our F2 hybrids show high transgressive values in behaviors related to sand sifting, and may thus have the potential to occupy a novel feeding niche.

## 2 MATERIALS AND METHODS

### 2.1 The F2 hybrids and their parental species

**FIGURE 1:**
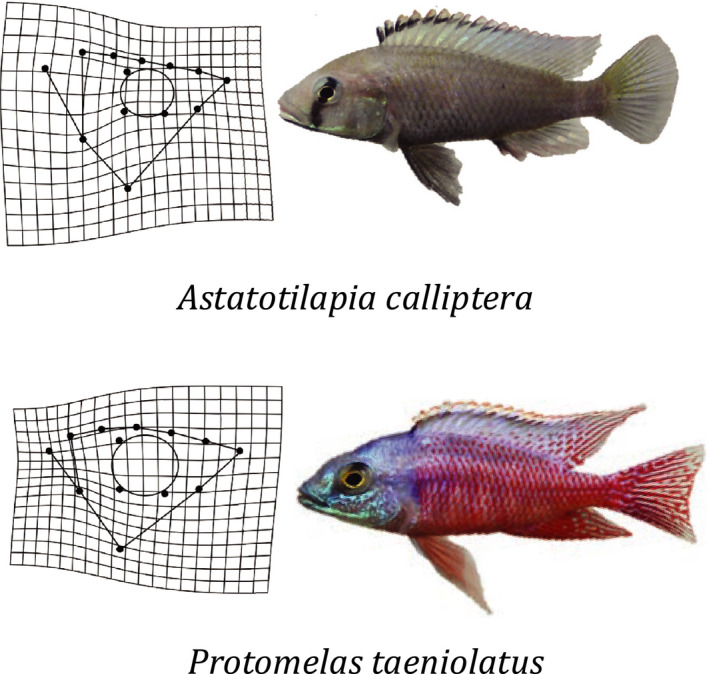
The two species used for the experimental cross. Shown are color photographs of representative male individuals of both species, *Astatotilapia calliptera* (CAL, top) *and Protomelas taeniolatus* (TAE, bottom). To the left of both: mean shape of TAE (ref) versus mean shape of CAL (target), and mean shape of CAL (ref) to mean shape of TAE (target), as plotted in geomorph (Adams & Otárola‐Castillo, 2013) using the plotRefToTarget function. Shape changes are 1.5× magnified

One of the parental species, *Astatotilapia calliptera*, henceforth referred to as CAL, is an omnivorous habitat generalist (Konings, 2010). Here, we used CAL from Chizumulu, an offshore island in Lake Malawi. The other parental species, *Protomelas taeniolatus,* henceforth referred to as TAE, is a generalist rock‐dwelling species from Lake Malawi, feeding mainly on loosely attached algae and the meiofauna living among the algae (Konings, 2010). See Figure 1 for a picture of both parental species and their morphologies.

Two half‐sib F1 hybrid families were obtained by crossing two CAL females with one TAE male. The six F2 hybrid families used in this study were obtained by crossing four males of one of the F1 families with five females of the same F1 family, and with one female of the other F1 family. Breeding protocols followed (Stelkens et al., 2009) and (Selz, Lucek, et al., 2014). All parental individuals used for breeding were derived from laboratory‐bred stock populations.

CAL individuals used for phenotyping and genotyping were also all derived from our stock population. Because only very few TAE individuals were available from our stock population, we added TAE individuals obtained from two aquarium breeders for phenotyping and genotyping.

We also included three females of specialized sand sifting species from Lake Malawi in the behavioral experiments: one *Otopharynx tetrastigma* female from our stock population, one *Taeniolethrinops* sp. female and one *Lethrinops* sp. female from the aquarium trade.

All fish were maintained and bred in a large recirculation aquarium system, with water temperature at 24–26°C and a 12:12 hr light/dark cycle. The fish were fed flake food once a day and a custom mix of spinach, ground shrimps, spirulina powder, and vitamins once a week.

### 2.2 Sand sifting behavior trials

**FIGURE 2:**
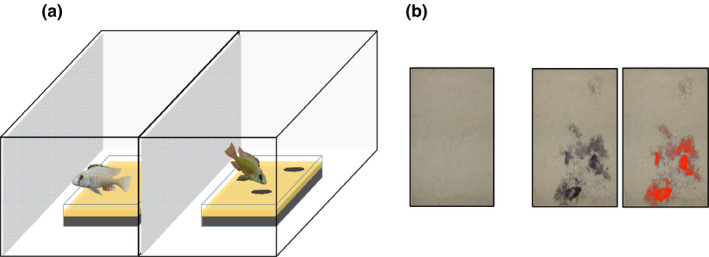
Setup for sand sifting behavior trials. (a) A tank that was part of a large recirculation aquarium system was divided up into eight adjacent compartments (only two shown) by metal plates with approx. 1 × 1 cm holes to ensure water flow. The compartments (78 × 36 cm) had a water depth of 32 cm and each contained a glass form (35 × 18 × 6 cm) which was filled with two layers of sand of different color. Where sand sifting occurred, black sand from the black bottom layer appeared on the beige sand surface (indicated as black spots on the right). Efficiency in sand sifting was calculated as the area of sand that had turned black divided by the number of strikes (how many times a fish picked up sand). (b) Visualization of color thresholding of sand pictures in ImageJ to measure the % of sand area that had been turned over—and hence turned black—in sand sifting trials. The same sand surface is shown before a trial (left) and after a trial without and with color threshold (right)

We used the setup shown in Figure 2a to assess frequency of and efficiency in sand sifting in the F2 hybrids and both parental species. The experimental procedure was slightly different for males and females. We observed spontaneous sand sifting in our stock tanks, independent of us feeding the fish. Thus, to avoid adding any confounding factors to our assay, we did not bury food in the sand for motivational purposes.

Each male fish was individually placed into one out of eight adjacent compartments (78 × 36 × 32 cm) separated by perforated barriers to ensure water flow (see Figure 2a). Each compartment contained a glass form (35 × 18 × 6 cm), which was initially left empty and then filled with two different layers of sand after the fish were acclimatized overnight (18 ± 2 hr). The bottom layer of sand consisted of 500 ml black sand with a grain size of 0.5–0.8 mm. The top layer consisted of 250 ml of beige sand with a grain size of ~1 mm, fully covering the black sand by a thin layer. The compartments themselves did not contain any sand and were covered from below with brown paper to prevent any light from shining through. A picture of the undisturbed sand was taken before and after the sand sifting experiment, which lasted for 2 hr and was filmed from the top as well as from the front of each compartment. For filming from the top and for the pictures, we used GOPRO HERO cameras (V 01.09, resolution 1080‐30). For filming from the front, we used a surveillance video system (Digi‐Protect, ABUS group security center). After the experiment, the fish and the sand were removed. Each fish was tested three times (with five exceptions as we lost some individuals before the experiments were completed), always with a different neighbor and/or in a different compartment. Three to eight (mode = 6) fish were tested simultaneously in adjacent compartments. Before the first trial and between trials, the fish were kept in single compartments (divided by a thin mesh from compartments containing other fish) for a minimum of 2 weeks to give the fish time to recover between trials and to minimize potential learning effects. Both the test and the single compartments were located in tanks that are part of the same large recirculation aquarium system where the fish were maintained (see above). Hence, the fish always had visual and olfactory contact with each other and/or with other fish. A total of 25 F2 hybrid males, 8 CAL, and 9 TAE males were tested with this setup.

The females showed signs of discomfort when tested individually. Hence, we adjusted the protocol as follows: Instead of just one, we placed three fish of one genotype class (CAL, TAE, F2 hybrids; henceforth “class”) together in a compartment, which allowed them to shoal (Pitcher, 1982), as we had observed them usually do in the stock tanks. To be able to distinguish the individuals visually, we sedated the females with MS222 (~75 mg/L, plus ~0.5 g/L sodium bicarbonate as buffer) and marked them with the ablation of a small part of fin (in the soft ray part either on the dorsal fin, on the upper or on the lower caudal fin lappet) one day before the first trial. No behavioral differences were observed between the marked and untreated females from the same tank. The compartments already contained the sand layers when we added the fish. The sand remained covered with a plastic mesh for an acclimatization time of three hours. Then we lifted the cover, took a picture of the sand, and started filming for 90 min from the front using the GOPRO HERO cameras. Afterward, another picture of the sand was taken and the fish were removed. Each group was tested twice, with a break of one day in between. 16 F2 hybrid groups, six CAL groups, and five TAE groups were tested with this setup. Two to six such groups were tested simultaneously in adjacent compartments. As activity visibly decreased from first to second trial, only the first trial per group was included in further analyses.

For a qualitative comparison with specialized sand sifting species from Lake Malawi, we also tested one group composed of one *Otopharynx tetrastigma* female, one *Taeniolethrinops* sp. female, and one *Lethrinops* sp. female in the same setup.

### 2.3 Analysis of male sand sifting trials

For each male trial, we measured and scored six different behaviors (Table 1). All behaviors were scored by the same person (AFF). Trials with “no strike” counts combined with a “hiding” or an “interaction” score of 5 (see Table 1) were excluded from analyses (10 out of 118 trials).

**TABLE 1:** Scored behaviors in male sand sifting trials

Scored behavior	Description
Strikes	Number of strikes, that is picking up of sand
Latency	Minutes to first strike
Scraping	Number of times scraping behavior was observed, that is a quick movement of scraping a part of the body on the sand
Scrape effect	Disturbance of sand surface after scraping: yes/no If yes, qualitatively categorized as follows: nearly all black (turned over sand) in one trial due to scraping: 0.9 a lot of black due to scraping: 0.75 half of black due to scraping: 0.5 some black due to scraping: 0.25 little black due to scraping: 0.1
Hiding	Qualitative score of the amount of time spent hiding or not moving (ranges from 1 to 5; 1 = no hiding, 5 = always hiding)
Interaction	Qualitative score of the amount of time spent interacting/displaying with/to neighboring fish (ranges from 1 to 5; 1 = no interaction, 5 = always interacting)

The pictures of the sand tray before and after each trial were imported into ImageJ I.49v (Rasband, 2015) as a sequence and then cropped so the pictures only contained the sand area. We color‐thresholded the pictures using the default thresholding method. The brightness level in the color threshold option was set to 80, which was the level we determined as best capturing the contrast between the black and beige sand particles (see Figure 2b). We then used the “analyse particles function” to calculate the percentage of area covered by black sand, that is, the area where black sand had been brought to the surface by the fish picking at or sifting through the sand (see Figure 2b). The score from the before‐trial picture was subtracted from the after‐trial picture. To remove effects of “scraping” (see Table 1), the score was multiplied by the corresponding inverse value of the effect due to scraping (see Table 1).

As a measure of efficiency, we divided the percentage of sand area that had been turned over by the number of strikes per individual (+0.001, to allow log‐transformation). This could only be calculated for trials in which at least one strike had occurred (73 out of 108 trials).

In the classical view, transgressive values in the F2 hybrids are defined as values outside the combined parental range (Parsons, Cooper, & Albertson, 2011; Rieseberg et al., 1999; Stelkens et al., 2009). In addition to this classical definition of transgression, we use more conservative transgression thresholds following (Parnell, Hulsey, & Streelman, 2012). Thus, we calculated transgression thresholds by taking the higher mean parental value plus two standard deviations (*SD*) for the high threshold, and the lower mean parental value minus two *SD*s for the low threshold. This approach also accounts for unequal sample sizes, as the lower sample size of the parental species should increase the *SD* and with it the thresholds for transgression (in both directions).

To do pairwise comparisons of means in strike counts and efficiency between the three classes (note, however, that population means are not predicted to change when transgressive variation occurs), we used the permTS function implemented in the perm package in R, with two‐sided tests using the exact.mc method and a conf. level of 0.95 (Fay & Shaw, 2010; R Development Core Team, 2015). For the measure of efficiency, this analysis was done for the raw (log10) values as well as for size‐corrected values given by the residuals of a linear regression (lm function) of (log10) efficiency versus (log10) standard length (SL) within each group to account for effects of size.

### 2.4 Analysis of female sand sifting trials

For every individual, we recorded latency and we counted the number of strikes and scraping behavior (as defined in Table 1). Black sand area and efficiency were calculated as described above, but using the summed‐up number of strikes of all three individuals in a group to calculate efficiency. Hence, the efficiency measure for females applies to the group and not to a single individual. This was possible for all groups except one group of TAE, which had zero strikes.

Additionally, the level of aggression within each group was qualitatively assessed (scores 1–5). Calculation of transgression thresholds and statistical comparisons of means were performed as outlined above.

### 2.5 Linear and geometric morphometrics

All fish used in experimental trials (*n* = 123) were anesthetized and subsequently euthanized in MS222 (~75 mg/L for anesthesia, ~200 mg/L for euthanizing, and ~0.5 g/L sodium bicarbonate added as buffer) and subsequently stored in 75% ethanol. Another 88 F2 hybrid individuals that had died of natural causes in the aquarium between the years 2010–2016 and that had been preserved in 75% ethanol were also available for morphological analysis. A standardized photograph was taken from the left side of each fish (161 F2 hybrids, 26 CAL, 24 TAE in total; including all individuals used in the behavioral trials) using a CANON E05 60D camera with a CANON 50 mm macro lens.

**FIGURE 3:**
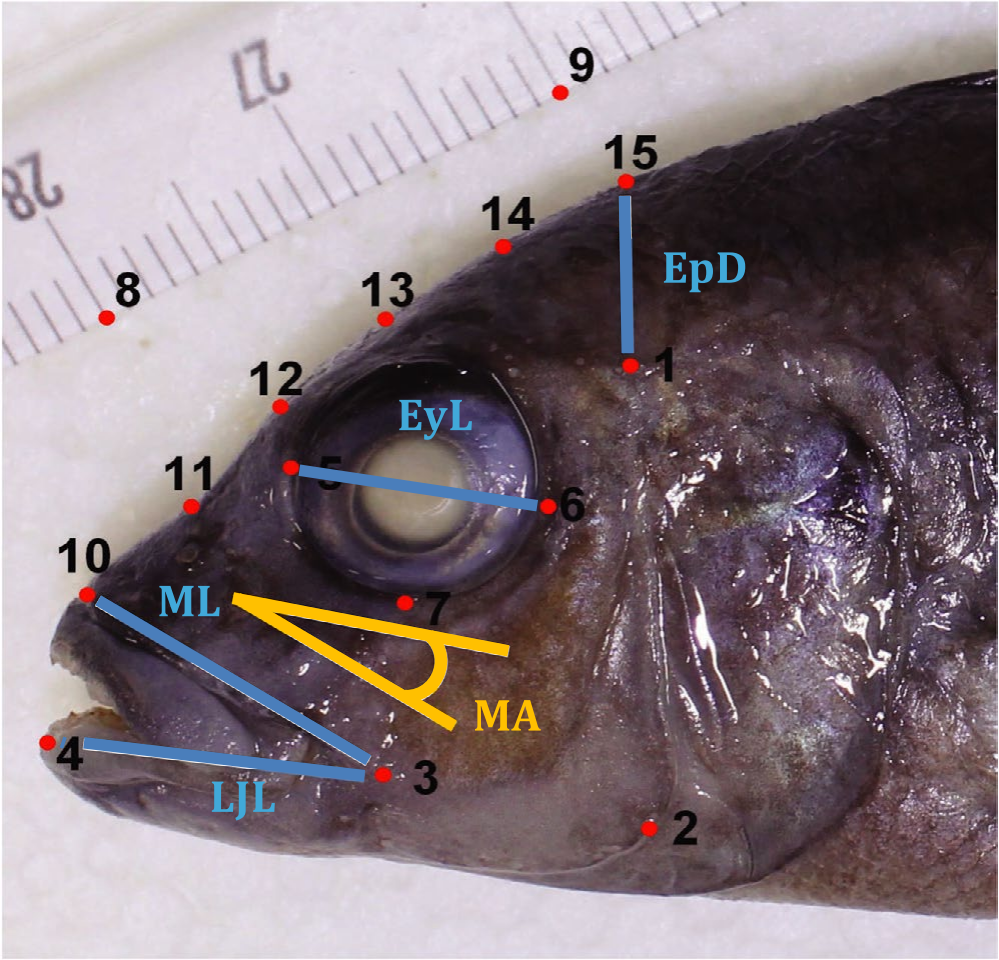
Morphological traits. Locations of landmarks (red) and the calculated linear distances (blue). Landmark locations: (1) intersection of scaled area and dorsal end of preoperculum; (2) ventral–posterior extreme at the bending point of preoperculum; (3) posterior hinge of lower jaw; (4) anterior tip of lower jaw; (5/6) anterior/ posterior extremes of eye socket, placed such that the line between them is parallel to the one between landmarks (2) and (3); (7) ventral extreme of eye socket; (10) anterior extreme of snout bone; (15) dorsal margin of the head above (1) vertical to horizontal body axis. (11–14) are semi‐landmarks equally spaced between landmarks (10) and (15). Linear distances: epaxial depth (EpD; 1,15), lower jaw length (LJL; 3,4), maxilla length (ML; 3,10), and eye length (EyL; 5,6). Standard length (SL; distance between the anterior extreme of snout bone and the caudal border of hypural plate at the lateral line) and head with (HW; distance between the posterior margins of the left and right operculum) were measured directly on the fish. Mouth angle (MA) was calculated as follows in R: (atan2((Y6‐Y5),(X6‐X5))*(180/π))‐(atan2((Y3‐Y10),(X3‐X10))*(180/π)). An upturned mouth will have a larger angle than a downturned mouth

In TPSdig 2.10 (Rohlf, 2010), we placed nine landmarks and four semi‐landmarks on each fish for morphometric analyses (Figure 3). The landmarks were set in such a way that they would be largely insensitive to slight head elevation due to lower jaw rotation, which we frequently encountered in the fish that had died of natural causes. The landmarks were used for geometric morphometric (shape) analysis and for calculating four linear distances as well as mouth angle (MA; Figure 3). The linear distances were Epaxial Depth (EpD), Eye Length (EyL), Lower Jaw Length (LJL), and Maxilla Length (ML) (Figure 3). Standard Length (SL) and Head Width (HW) were directly measured on the fish (average of three measurements with an accepted error of max. 5%). These traits were chosen to capture aspects of functional morphology associated with the generation of high suction pressure (McGee, Schluter, & Wainwright, 2013) without the need to clear and stain the specimens, which was not possible due to constraints on specimen use for other projects. All analysis of morphological data was performed in R, using the R package geomorph (Adams & Otárola‐Castillo, 2013).

Size correction of all scaled linear traits was performed by taking the residuals of a linear regression of the respective (log_10_‐transformed) trait values against (log_10_‐transformed) SL. As the slopes of the three classes were homogeneous for all these traits (ANOVA, FDR‐adjusted *p*‐values > .1; before FDR correction two traits showed nonhomogenous slopes: EyL [*F*_2,205_ = 3.361, *p* = .04*] and ML [*F*_2,205_ = 3.192, *p* = .04*]), size correction was performed for all three classes simultaneously. We calculated transgression thresholds as described above (following Parnell et al., 2012).

We additionally performed a principal component analysis (PCA) including all size‐corrected linear traits and MA to compare occupation of morphospace of the F2 hybrids and both parental species.

To remove an effect of open mouth (see above) in geometric morphometric analysis, we rotated landmark 4 such that the angle between ML and LJL was the same for all specimen (mean angle over all specimens; (Adams, 1999). To obtain shape data, the landmark configurations were subjected to a generalized Procrustes analysis (GPA). During superimposition, the semi‐landmarks (see Figure 3) were slid to minimize bending energy (Bookstein, 1997). As shape variation was significantly associated with SL due to allometric effects (Procrustes ANOVA: *F*_1,209_ = 41.962, *p* = .001**), we took the residuals of a linear regression of shape coordinates on (log_10_) SL, and added them to the consensus coordinates to obtain allometry‐free shapes (Adams & Otárola‐Castillo, 2013; Klingenberg, 2016). Homogeneity of slopes of the F2 hybrids and both parental species was given (ANOVA with randomized residual permutation procedure: *F*_205_ = 0.716, *p* = .77), hence size correction was performed over all three classes (F2 hybrids and both parental species) simultaneously. The major axes of shape variation were then assessed using principal component analyses (PCA) and plotted as morphospace with warpgrids (visualizing the difference between mean shape and shape at the maximum and minimum end, respectively, of a PC axis) for visual inspection of shape change along PC axes. We calculated transgression thresholds for each axis as described above (following (Parnell et al., 2012)).

Additionally, we performed the PCA only including the F2 hybrids—these PC scores were subsequently used to test for associations with efficiency in males and in QTL mapping—and we then used the predict function to project the parental species into this morphospace.

For shape comparisons with specialized sand sifting species, we placed the same set of landmarks on photographs of 21 preserved specimens (of samples stored at the Natural History Museum [London, UK], Africa Museum [Tervuren, Belgium], Naturalis Museum [Leiden, Netherlands] and the collections of OS) representing 21 different sand sifting species from Lake Malawi (see List in Appendix S1 and Young, Snoeks, & Seehausen, 2009). On the pictures of these fish, we added an additional landmark on the caudal border of the hypural plate at the lateral line to calculate SL (distance between this additional landmark and landmark 10, see Figure 3). While angle correction and Procrustes analysis (GPA) were performed on the whole extended dataset, size correction for these individuals was performed separately as they were generally larger and had significantly different allometry slopes (*F*_224_ = 0.02, *p* = .01*). We then used the predict function to project the sand sifting species into the morphospace occupied by the F2 hybrids and both parental species.

### 2.6 Associations between efficiency and morphology in F2 hybrid males

Associations of sand sifting efficiency with morphological traits in the F2 hybrid males were tested using linear models (lm function in R) with maximum sand sifting efficiency as response variable. First, we tested each trait separately, i.e. all linear traits, MA, PC scores of all linear traits including MA (of the PCA including only F2 hybrids), and shape PC scores (of the PCA including only F2 hybrids). All linear traits (including MA) together, all linear PC scores together, and all shape PC scores together were then also tested using multiple linear models, using the drop1 function and likelihood ratio tests (ANOVA function) for backward model selection, and basing selection of the final model on (lowest) AIC score. For model validation, residuals were checked for normality (qqplots implemented in the car package; Fox & Weisberg, 2011) and heteroscedasticity by plotting them against fitted values. All p‐values were adjusted for FDR (Benjamini & Hochberg, 1995; Verhoeven, Simonsen, & McIntyre, 2005).

### 2.7 RAD sequencing

Finclips of all individuals used in behavioral and morphological analyses (see overview on Datadryad) for RAD‐tag sequencing had been stored in 98% ethanol. DNA was extracted from finclips using a phenol–chloroform protocol (Sambrook & Russell, 2001). We prepared four RAD libraries following Baird et al. (2008), with some modifications. We used 1 μg genomic DNA per sample. Restriction digestion was performed overnight using the restriction endonuclease HF‐*Sbf*I (NewEngland Biolabs). We multiplexed 48 individuals per library after the ligation step using TruSeq P1‐adapters and custom 5bp‐8bp barcodes. Each library contained 5–18 F2 hybrid males, 14–23 F2 hybrid females, 5–6 F2 hybrid individuals of undetermined sex, and 3–11 parental species individuals (of both sexes). The libraries were sheared for two minutes on a COVARIS M220 Focused‐ultrasonicator (Covaris Inc. 2012). Sheared fragments between 300 and 700 bp were selected on a SageELF machine (Sage Science Inc. 2014). All libraries were single end‐sequenced (100 bp) on an Illumina HiSeq 2,500 platform (Illumina Inc 2012). Each library was sequenced on a single lane. 5%–12.5% bacteriophage PhiX genomic DNA was added to each lane.

We demultiplexed and trimmed the reads to 90 bp using the process_radtags script from Stacks v1.40 (Catchen, Hohenlohe, Bassham, Amores, & Cresko, 2013), correcting single errors in the barcode and the restriction site, and discarding reads with incomplete restriction sites. Using the FastX toolkit (http://hannonlab.cshl.edu/fastx_toolkit/index.html), we removed all reads with a Phred quality score below 10, and reads with more than 5% of bases with a quality score of less than 30. The reads of each individual were aligned to an anchored version of the *Pundamilia nyererei* reference genome (Feulner, Schwarzer, Haesler, Meier, & Seehausen, 2018). End‐to‐end alignment was performed with Bowtie2 (Langmead & Salzberg, 2012) using default parameters. Base score recalibration was performed as described in (Marques et al., 2016). GATK Unified Genotyper v3.7 (McKenna et al., 2010) was used to call all confident sites (with a minimum base quality score of 20). Filtering was performed with a custom Python script and VCFtools v0.1.14 (Danecek et al., 2011). Minimum variant quality value was 30, genotypes were required to have minimum quality value of 30, and a minimum depth of coverage of 10 reads. Indels, sites within 10bp of an indel, multiallelic SNPs and monomorphic sites were removed. Additionally, sites with more than 50% missing data were removed, as well as sites with a mean genotype depth greater than 88 (median x1.5; as these are expected to be enriched for paralogs). Furthermore, singletons and doubletons were removed because they would not be informative for QTL mapping. Individuals with a mean depth below 20 and/or more than 50% missing data (*n* = 12) were excluded. Another 33 individuals (of which 20 belonged to the previously preserved F2 hybrids) were excluded due to potential PCR duplication and allelic dropout, which was assessed by plotting sequencing depth against minor allele reads and inspecting the proportion of minor alleles reads for each individual. Finally, we filtered out sites within 200 bp of each other to obtain one single SNP per RAD locus. The final vcf file contained 126 individuals (35 F2 males, 70 F2 females, nine F2s of undetermined sex, four CAL males, two CAL females, one CAL of undetermined sex, four TAE males, one TAE female) and 10,166 SNPs.

### 2.8 Linkage map and QTL mapping

As finclips of the grandparents of our cross were not available, we used multiple individuals of each parental population as a substitute (six CAL [three males, two females, one undetermined], five TAE [four males, one female]) to identify reciprocally fixed loci that were then used for mapping. No more than three out of the six CAL and two out of the five TAE were allowed to have missing data at a given SNP. The resulting set of 1,188 SNPs over 113 F2 individuals (34 males, 70 females, nine undetermined) was imported into JoinMap 4.1 (van Ooijen, 2006) to produce a linkage map. Premapping quality control resulted in excluding markers under severe segregation distortion (*p* < .01; *n* = 179) and exhibiting >20% missing data (*n* = 19), and in excluding individuals with >30% missing data (*n* = 2). Based on an independence, LOD threshold of 7.0 the markers were grouped into 22 linkage groups, corresponding to the expected number of chromosomes in these species (Simakov et al., 2014). Another 9 loci with SCL (Strongest Cross Link)‐Values above 5 were excluded in this process. For mapping, the regression algorithm and Kosambi's mapping function were used (LOD threshold 1.0, recombination threshold 0.499, goodness‐of‐fit jump threshold 5.0, no fixed order; two rounds of mapping, ripple performed after each marker addition). After map creation, markers with an unusually high nearest neighbor fit were excluded (*n* = 5). The resulting map contained a total of 931 markers. The mean number of markers per LG was 42.32 (*SD* = 11.98; min = 17, max = 64).

QTL mapping was performed using the scanone function (standard interval mapping using the method EM algorithm (Lander & Botstein, 1989) in the R/qtl package (Broman, Wu, Sen, & Churchill, 2003)). Conditional genotype probabilities were calculated using the calc.genoprob function with a step‐size of 1 cM, an error.prob of 0.05 and the Kosambi mapping function. Mapping was performed on all five linear traits and MA together, and on shape PC axes 1–6 together (axes explaining min. 5% of variance) using normal models. Sand sifting behaviors (maximum observed efficiency and number of strikes in males and number of strikes in females) were mapped separately using both normal and nonparametric models. Genome‐wide significance thresholds were determined by permutation (*n* = 1,000). This was done for all fish together without and with sex as covariate for the morphological traits. Bayesian credible intervals were calculated using the baysint function. Percent variance (PVE) was calculated as 1–10^−(2/^ *^n^* ^)*LOD^ following (Broman et al., 2003), where *n* is the number of individuals.

## 3 PERMITS

Fish experimentation and euthanasia were authorized by the veterinary office of the canton of Lucerne (License number: LU04/07).

## 4 RESULTS

### 4.1 Sand sifting behavior

#### 4.1.1 Transgression in sand sifting behaviors in some F2 hybrids

**FIGURE 4:**
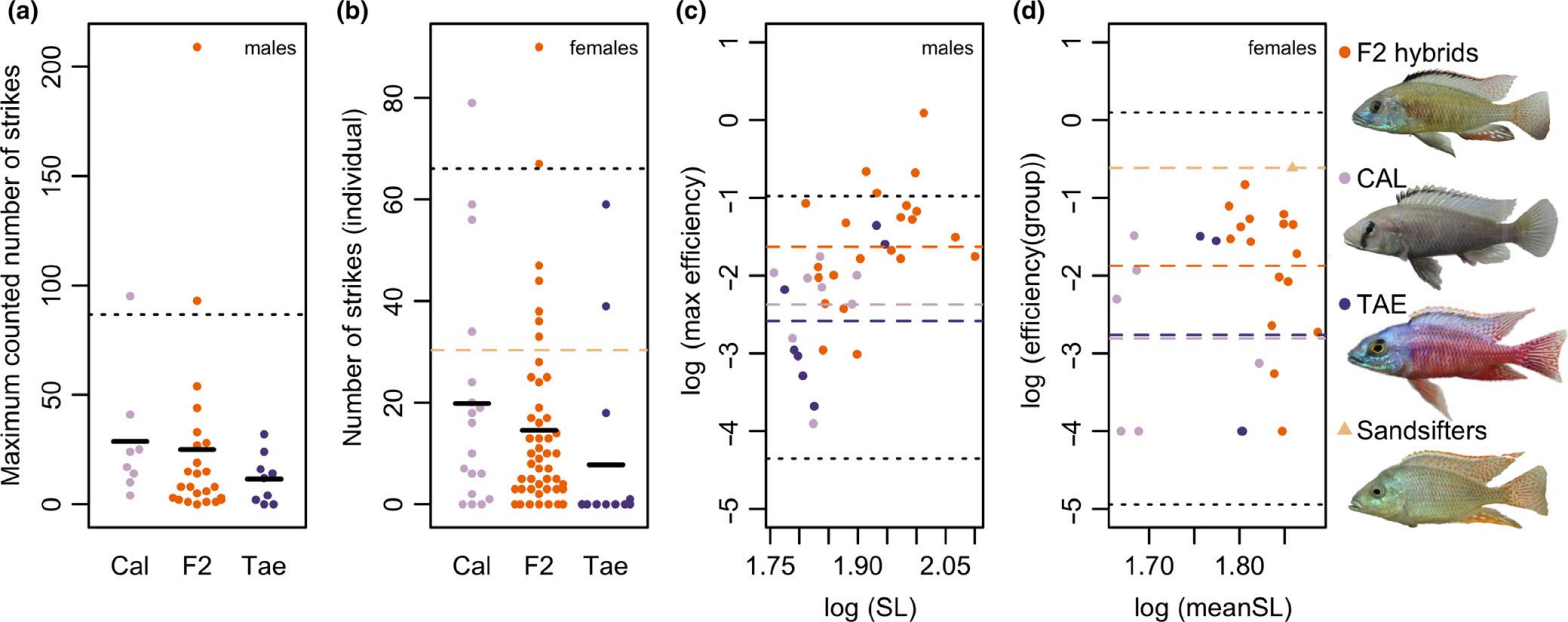
Some F2 hybrids have transgressive behavioral scores. (a, b) show strike counts (i.e., how often sand was picked up by an individual) in sand sifting trials. For males (a), the maximum observed number of strikes for each individual is shown (for per trial strikes and means over all three trials see Figure S1). For females, (b), the number of strikes for each individual in the first trial is shown. (c, d) show sand sifting efficiency (log10‐transformed) plotted against standard length (SL; log10‐transformed). For males (c), the maximum observed efficiency for each individual is shown (per trial efficiencies and means over all three trials shown in Figure S1). For females (d), efficiency per group in the first trial is shown. (SL here is also the mean of the three females in a group). Additionally, one group including three females of three different specialized sand sifting species is shown (“Sandsifters”). The fish shown on the right are male representatives of each of the three classes, and an *Otopharynx tetrastigma* male is shown as representative of one of the tested specialized sand sifting species. The black dotted lines in all plots indicate transgression thresholds given by the highest/lowest parental species mean +/‐ 2 standard deviations. The black bars in (a, b) indicate the means. The dashed lines with different colors indicate the mean for each class in (c, d), in (b) it indicates the mean of the sand sifting species group

When analyzing the number of strikes (i.e., how often sand was picked up), we found that some F2 hybrid males had transgressive values (in the positive direction). This outcome was similar when considering the maximum observed number of strikes per individual (Figure 4a), or when conservatively considering the mean number of strikes over all three trials, or the number of strikes per trial separately (Figure S1). One or two males of the CAL parental species also had high values, but they were not as high as the most extreme F2 hybrids. Permutation tests to compare the means of the F2 males and the males of the two parental species revealed no significant difference between any of the three classes in the maximum observed number of strikes (F2 vs. CAL *p* = .754, F2 vs. TAE *p* = .426, TAE vs. CAL *p* = .104).

Similar to the males, two of the F2 females had transgressive values (positive direction) in the number of strikes, as did one CAL female (Figure 4b). Permutation tests to compare the means in number strikes among the F2 females and the females of the two parental species revealed no significant differences (F2 vs. CAL *p* = .364, F2 vs. TAE *p* = .208, TAE vs. CAL *p* = .120).

In efficiency, some F2 hybrid males clearly exceeded the combined range of parental scores as well as the upper transgression threshold (Figure 4c). While several F2 hybrid males were considerably larger than parental species males, transgressive values were not limited to those individuals; that is, some F2 hybrid males within the size range of the parental species showed transgressive values for the behavior (in the positive direction). Again, this was similar when comparing maximum (Figure 4c) or per trial efficiency (with the exception of the third trial, which may indicate learning effects) (Figure S1). Permutation tests to compare the means in maximum observed efficiencies among the three classes revealed significant differences between the F2 hybrid and either parental species (F2 vs. CAL *p* = .022, F2 vs. TAE *p* = .012, TAE vs. CAL *p* = .528). However, these differences were no longer significant when including a size correction in the analysis (F2 vs. CAL *p* = .950, F2 vs. TAE *p* = .994, TAE vs. CAL *p* = .940).

While some F2 female groups had transgressive efficiency values (exceeding the combined parental range), F2 female groups were larger on average and none of them crossed the (upper) transgression threshold (Figure 4d). Permutation tests to compare the means in efficiency revealed a marginally significant difference between the F2 hybrid and CAL female groups (F2 vs. CAL *p* = .068, F2 vs. TAE *p* = .128, TAE vs. CAL *p* = .998). However, there were no significant differences among the three female classes when including a size correction in the analysis (F2 vs. CAL *p* = .994, F2 vs. TAE *p* = .994, TAE vs. CAL *p* = .988).

The mixed group of sand sifting species had a higher overall strike count (dashed brown line in Figure 4b) and as expected, they had a higher sand sifting efficiency score than any of the other female groups (Figure 4d).

### 4.2 Morphology

#### 4.2.1 Transgression in several linear traits

**FIGURE 5:**
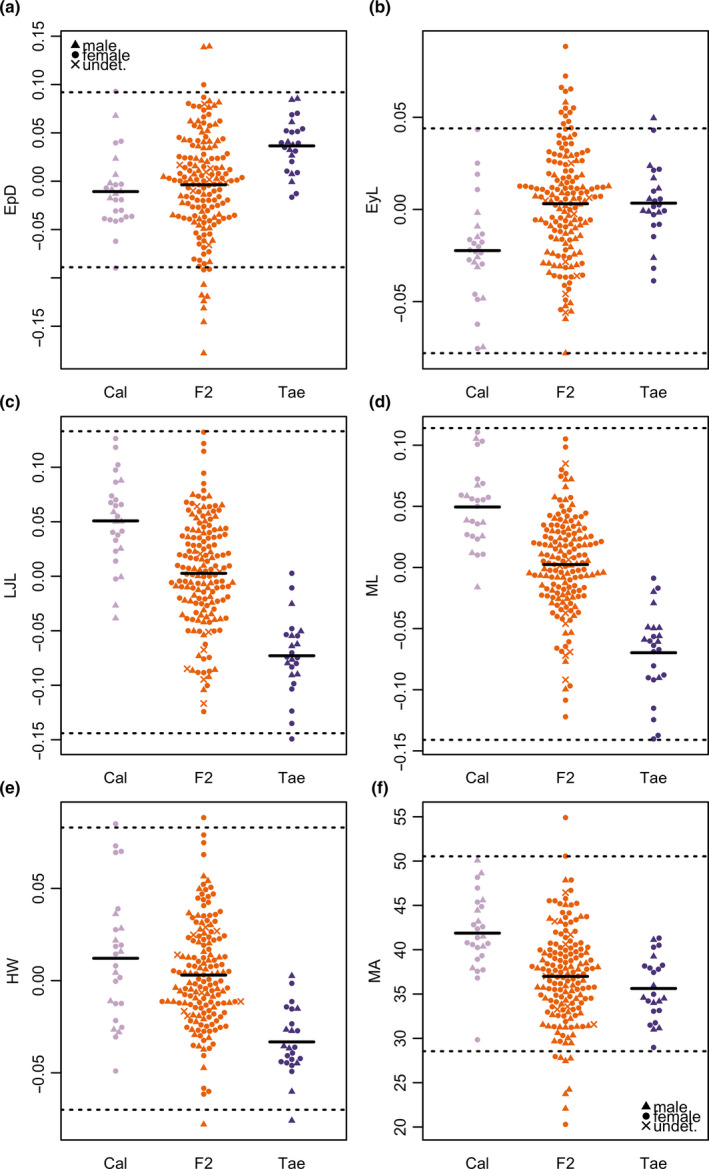
Some F2 hybrids have transgressive morphological trait values. Distributions of the five measured (and size‐corrected) linear traits and Mouth Angle (MA) for all three classes, including males and females (different symbols). (a) Epaxial Depth (EpD) (b) Eye Length (EyL) (c) Lower Jaw Length (LJL) (d) Maxilla Length (ML) (e) Head Width (HW) (f) Mouth Angle (MA). The black dotted lines indicate transgression thresholds given by the highest/lowest parental species mean +/‐ 2 standard deviations. The black bars indicate means in each class

Transgression in the F2 hybrids was observed in Epaxial Depth (EpD; both directions), Eye Length (EyL; positive direction), Mouth Angle (MA; both directions), and Head Width (HW; both directions) (Figure 5, Table S1). However, for HW, this only concerned one individual each, and for this trait, the transgression thresholds were still within the combined parental species’ range. There were some differences in the directionality of transgression when calculating the thresholds for males and females separately (see Table S1). Maxilla Length (ML) and to some extent Lower Jaw Length (LJL) differentiate the two parental species (Figure 5): CAL have larger values in both traits.

In the PCA on all linear traits and MA (Figure S2), some individuals fell outside transgression thresholds on several of the PC axes. Furthermore, the F2 hybrid multivariate morphospace (as 95% confidence interval ellipses) covered a larger area than either parental species’, and also covered areas outside both parental morphospaces on PC1 versus PC2, for example lower left and upper right areas (Figure S2a; and see Table S2 for trait loadings).

#### 4.2.2 Areas of novelty in shape

**FIGURE 6:**
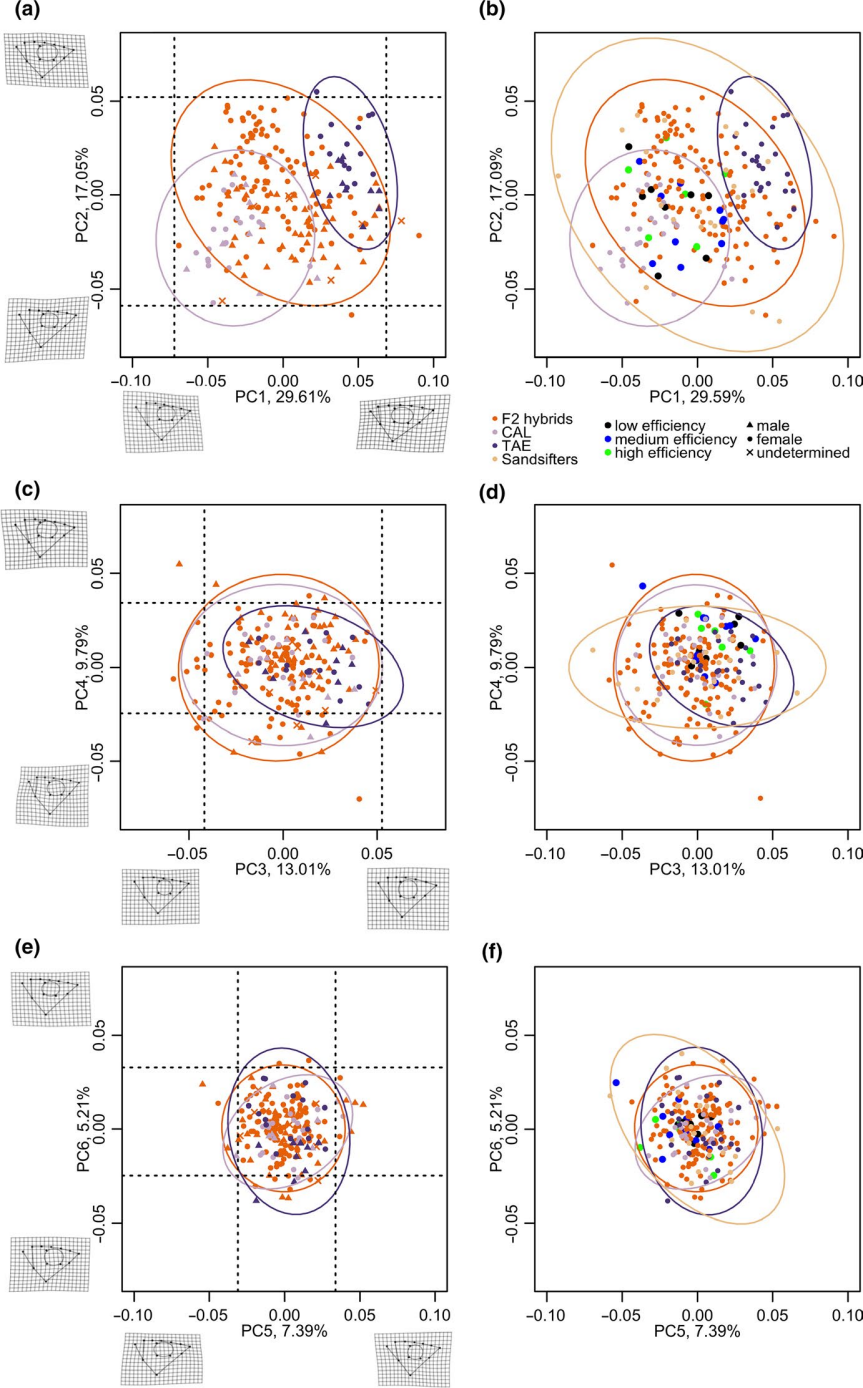
Some F2 hybrids are transgressive in shape and some occupy morphospace not shared with either parental species. Principal component analyses (PCA) of shape. (a) PC1 versus PC2, (c) PC3 versus PC4, (e) PC5 versus PC6. (b, d, f) feature the same PC axes as in (a, c, e) but here the dataset includes 21 sand sifting species (“Sandsifters,” one data point per species; see list in Appendix S1), which were projected into the morphospace made up by the F2 hybrids and both parental species. Additionally, F2 hybrid males that we tested in behavioral trials are highlighted according to their mean sand sifting efficiency (low, medium, high). The black dotted lines indicate transgression thresholds on a given axis given by the highest/lowest parental species mean value +/‐ 2 standard deviations. Ellipses represent 95% confidence intervals. Warpgrids show shape differences between mean shape and the most extreme shape at a given PC axis

In the analyses including the F2 hybrids and both parental species, PC1 and to some extent PC2 differentiated the parental species (Figure 6). Shape change along PC1 was mainly driven by mouth size and epaxial depth, such that TAE had a smaller mouth but a deeper epaxial muscle than CAL. Shape change along PC2 was mainly driven by eye size and the angle of the mouth, such that TAE had larger eyes and a more downturned mouth. As in the PCA of linear traits, some F2 hybrid individuals fell outside transgression thresholds on several PC axes (Figure 6). Also, the F2 hybrid morphospace (as 95% confidence interval ellipse) covered an overall larger area as well as areas outside both parental species morphospace on PC1 versus PC2 (Figure 6a). The most extreme transgression seemed to occur on PC4 (Figure 6c), where shape variation was mainly driven by mouth angle and mouth size: Individuals on the maximum end of this axis had a larger downturned mouth, while individuals on the minimum end of this axis had a smaller upturned mouth (Figure 6c). These are novel combinations compared to the parental species as CAL have a larger and more upturned mouth, and smaller eyes and a smaller epaxial depth compared to TAE (Figure 1).

When the morphospace of the 21 sand sifting species we measured was projected into the F2 hybrid and parental species morphospace, it encompassed the parental species morphospace as well as the transgressive morphospace of the F2s on PC1 versus PC2 (Figure 6b). This was mostly true also for PC3 (Figure 6d) and PC5 versus PC6 (Figure 6f), but on PC4 (Figure 6d), a number of F2 hybrids came to lie well outside the sand sifting species morphospace.

When shape morphospace was defined by the variance in the F2 hybrids and the individuals of the parental species were predicted into this morphospace (Figure S3; note that these were the PC values later used in QTL mapping), there was again a substantial area where the F2 hybrids occupied morphospace not covered by either parental species (lower right corner in Figure S3a). Compared to the minimum end (i.e., negative sign) of PC1, shape on the maximum end (i.e., positive sign) of PC1 was characterized by a large mouth (CAL‐like), a large eye (TAE‐like), small epaxial depth (CAL‐like), and a convex dorsal head profile (more TAE‐like). On PC2, shape on the minimum end was characterized by a rather shallow head with a long snout and a small downturned mouth (overall more TAE‐like). Hence, F2 hybrids in this morphospace outside both parental species morphospaces combined traits in a novel way.

#### 4.2.3 No significant associations of morphology with efficiency in F2 hybrid males

None of the linear traits or shapes were significantly associated with maximum scored sand sifting efficiency in males (all *p* > .1 after FDR correction; see Table S3). The best (but not significant) models with multiple traits based on AIC were a model containing shape PC axes 4–6, in which PC4 and PC6 had only marginally significant effects (*p* = .05 and *p* = .06 before FDR correction), and a model containing EyL, LJL, and ML, in which ML (a nontransgressive trait) had an only marginally significant effect (*p* = .08 before FDR correction) (see Table S3). Also, the behaviorally tested F2 hybrid males did not cluster by efficiency category (low, medium, high mean efficiency) in the shape PCA shown in Figure 6b,d,f.

### 4.3 QTL Mapping

#### 4.3.1 A marginally significant QTL for sand sifting

**FIGURE 7:**
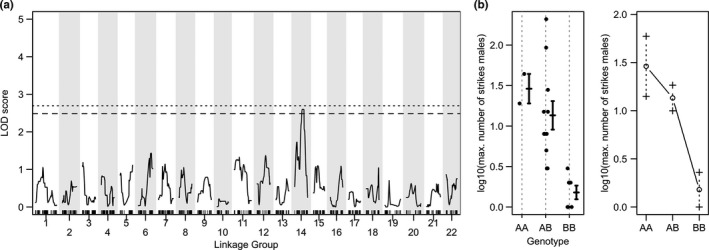
A marginally significant QTL for sand sifting frequency in males. (a) LOD scores across the 22 chromosomes for the maximum observed number of strikes in F2 hybrid males. The dotted line represents a genome‐wide significance threshold of *p* < .05, the dashed line of *p* < .1. (b) shows the phenotypic distribution (log10 transformed) and effect plots for this trait at the most significant marker associated with the QTL on LG14. Genotype AA corresponds to homozygous for CAL alleles, BB to homozygous for TAE alleles, AB to heterozygous

We found one marginally significant QTL for the number of strikes in males on LG14 (Pun‐LG22/Ore‐LG12 (*p* = .072; Table 2, Figure 7) using the nonparametric model in mapping. Homozygotes for the CAL allele had the highest scores, homozygotes for TAE the lowest.

**TABLE 2:** Overview of significant and marginally significant QTLs

TRAIT	Marker (nearest)	LG	Pun‐LG	Ore‐LG	cM	95% CI (cM) (nearest markers)	LOD	*p*‐Value	PVE
*All fish (n = 107), no covariates*								
PC2 (shape)	c22.loc29 (chr14_13840517)	22	14	9	29	17.7–44.9 (chr14_18035646‐scaffold_539_97894)	4.23	**.026**	16.64
PC2 (shape)	chr17_2791538	10	17	20	0.42	0–36.2 (chr17_1473009‐chr17_27424319)	3.67	.067	14.61
*All fish (n = 103), sex as additive covariate*								
PC2 (shape)	chr17_2791538	10	17	20	0.42	0–36.2 (chr17_1473009‐chr17_27424319	3.53	.077	14.60
*All fish (n = 103), sex as additive + interactive covariate*								
EpD	scaffold_600_85054	17	6	11	5.32	0–20.6 (chr6_2259192‐chr6_13717514)	7.79	**.021**	29.41
PC2 (shape)	chr20_123196	13	20	4	56.2	24.8–56.2 (chr20_10993627‐chr20_123196)	5.02	**.038**	20.10
*Males (n = 19)*								
Number of strikes	c14.loc34 (chr22_9564051)	14	22	12	34	0–49.04 (chr22_23331169‐scaffold_202_1018989)	2.60	.072	46.75

Note

Nearest, nearest marker to a QTL at an interpolated marker where genotypes were inferred with calc.genoprob; Pun‐LG, linkage group number corresponding to the anchored *Pundamilia nyererei* reference genome (Feulner et al., 2018); Ore‐LG, linkage group number corresponding to the *Oreochromis niloticus* reference genome (Simakov et al., 2014); 95% CI Bayesian 95% confidence interval; cM, position in centi Morgan; PVE, percent variance explained, calculated as 1–10^−(2*/^ *^n^* ^)*LOD^ following (Broman et al., 2003).

#### 4.3.2 Significant QTLs for Epaxial Depth (EpD) and shape PC2

**FIGURE 8:**
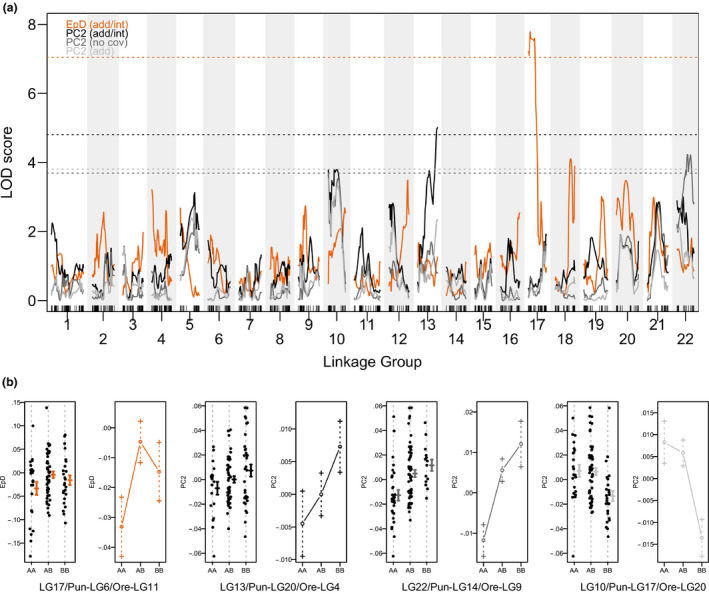
QTL mapping suggests the presence of some loci with moderate to large effects on morphology. In (a) LOD scores across the 22 chromosomes are shown for EpD and PC2 (mapping with sex as additive (add)/interactive(int) covariates for EpD and PC2, and without covariates (no cov) for PC2), for which we found significant (*p* < .05) or marginally significant (*p* < .1) QTLs. The dotted lines represent genome‐wide significance thresholds of *p* < .05. (b) shows the phenotypic distributions and effect plots of these two traits at the most significant marker associated with a QTL. Genotype AA corresponds to homozygous for CAL alleles, BB to homozygous for TAE alleles, AB to heterozygous

We found one significant QTL for EpD (*p* = .021; Table 2, Figure 8) explaining 29.4% of variance on LG17 (Pun‐LG6, Ore‐LG11) when accounting for sex as additive and interactive covariate (allows average phenotype as well as QTL effect to be different between the two sexes). At the most significant marker for this QTL, homozygotes for the CAL allele had a smaller EpD than homozygotes for the TAE, and heterozygotes had the largest values (Figure 8b).

We found two significant QTLs for shape PC2: one on LG22 (Pun‐LG14, Ore‐LG9) explaining 16.6% of variance in the mapping without sex as covariate, and one on LG13 (Pun‐LG20, Ore‐LG4) explaining 20.1% of variance, when accounting for sex as additive and interactive covariate (*p* = .038; Table 2, Figure 8). At the most significant markers for these QTLs, homozygotes for the CAL allele had a low PC2 score and homozygotes for the TAE allele had a high PC2 score in both cases (Figure 8). We found another marginally significant (*p* < .1) QTL for PC2 on LG10 (Pun‐LG17, Ore‐LG20) explaining 14.6% of variance, either when mapping without covariates, (*p* = .067), or when accounting for sex as additive covariate (*p* = .077) (Table 2, Figure 8). At the most significant markers for this QTL, homozygotes for the CAL allele had a high PC2 score and homozygotes for the TAE allele had a low PC2 score.

## 5 DISCUSSION

We experimentally investigated transgressive segregation in morphology and behavior that may result in the potential to exploit a novel ecological niche (i.e., sand sifting) in a second‐generation (F2) hybrid cross between two trophic generalist species of Lake Malawi cichlids, in which some of the hybrids phenotypically (in body shape and color) resembled sand sifting species in the adaptive radiation of Lake Malawi cichlids (Fryer & Iles, 1972; Konings, 2010).

Our experimental trials revealed that F2 hybrid males displayed sand sifting behavior at similar mean frequencies as males of both parental species. However, a few F2 hybrid individuals were transgressive: they came to lie outside and above the combined parental species range and they exceeded the upper transgression threshold given by the higher parental species mean + 2 *SD*. Similarly, mean sand sifting efficiency scores were similar among the three classes after accounting for size. Yet again, some hybrid males were transgressive, clearly positively exceeding the combined parental species range as well as the upper transgression threshold. This also applied to some hybrid individuals within the size range of parental individuals; hence, size alone does not seem sufficient to explain high efficiency. F2 hybrid females displayed sand sifting behavior at similar mean frequencies as females from both parental species. While two F2 individuals had transgressive scores, this also applied to one CAL female. Some hybrid female groups had efficiency scores outside and above the parental species range, but not crossing the transgression threshold (and the F2 females were overall also larger than the parental species females). However, some of these F2 hybrid female groups reached efficiency values close to the high value of the one group of specialized sand sifting species that we tested. Hence, we observed rather extreme functional trait values related to a specialized mode of feeding on a resource that neither parental species typically exploits. Sand sifting could also be related to behaviors other than feeding, for example, bower building (McKaye, Stauffer, Turner, Konings, & Sato, 2001). Interestingly, most of the specialized sand sifters among Lake Malawi cichlids are also male bower builders (Fryer & Iles, 1972; York, Patil, Hulsey, Streelman, & Fernald, 2015). So evolutionary interactions between the two functions (feeding and reproduction) may have played a role in the adaptive radiation. Bower building has not been observed in either parental species of our cross. We think we can exclude a (transgressive) motivation for bower building to explain our data because such behavior would only be expected in reproductively active males and usually only well after they have established a territory (Fryer & Iles, 1972; York et al., 2015).

Second, we found individuals among the hybrids with transgressive values in several linear morphological traits. As expected from theory (Stelkens & Seehausen, 2009) and demonstrated in cichlids (Stelkens et al., 2009), transgression occurred predominantly in traits that do not differentiate the parental species. In our multivariate principal component analyses, where each axis is built from different combinations of variance components of the morphometric traits, we observed some transgression on individual axes. Moreover, we found areas where the F2 hybrids occupied distinct parts of morphospace not covered by either parental species through novel combinations of parental trait values on different shape axes (i.e., areas of phenotypic novelty). Hence, we show that hybridization can rapidly generate transgressive morphology and novel morphological trait combinations, which may provide the raw material for rapid adaptive diversification (Grant & Grant, 1992; Lewontin & Birch, 1966; Stebbins, 1959).

The absence of any simple significant association between morphology and sand sifting efficiency likely reflects the complexity of the functional relationships associated with the behavior (Kane & Higham, 2015). The functional basis of benthic foraging on single targeted prey items is relatively well understood (McGee et al., 2013), but the factors governing more indiscriminate sifting of large quantities of sand are more complex. Suction potential can be enhanced via enlarged epaxial musculature or a reduction in buccal volume (McGee et al., 2013), which can be seen in one of our parental species, *P. taeniolatus* (TAE), which has both a small gape and enlarged epaxial musculature relative to *A. calliptera* (CAL) (see Figure 1). However, while a smaller buccal volume may enhance suction, it also limits the amount of substrate processed per strike, compromising efficiency. This suggests that sand sifters may experience a trade‐off between suction potential and sifting efficiency, producing an adaptive ridge in morphospace where no one trait combination is optimal. Furthermore, other behavioral aspects that we did not measure in our experiment, such as at which angle a fish approaches the sand, may influence efficiency in sand sifting.

We found significant QTLs for one linear trait (Epaxial Depth, EpD) and for shape PC2. The QTL for EpD could to some extent be predicted to generate transgressive phenotypes via overdominance since heterozygous genotypes had the highest values in this trait (Figure 8b). However, although three individuals were indeed transgressive in this direction for this trait (Figure 5a), more individuals were transgressive in the opposite direction, and these low values occurred in homozygous CAL genotypes, indicating that other mechanisms, such as epistasis, could be involved in generating transgression in this trait. On shape PC2, the more deep‐headed individuals with a large upturned mouth and short snout (more CAL‐like) were on the maximum end of PC2, the more slender‐headed individuals with a small downturned mouth and a longer snout (more TAE‐like) were on the minimum end of PC2 (see Figure S3a). The genotypes at the marginally significant QTL on LG10 (Pun‐LG17/Ore‐LG20) are consistent with this: individuals that are homozygous for CAL alleles (AA in Figure 8b) had higher PC2 values while individuals homozygous for TAE alleles had lower PC values. The opposite is true for the other two (significant) QTLs for shape PC2 (one on LG13/Pun‐LG20/Ore‐LG4 and one on LG22/Pun‐LG14/Ore‐LG9), where individuals homozygous for CAL alleles had lower and individuals homozygous for TAE alleles had higher PC2 values (Figure 8b). Position on PC2 hence has a genomic architecture as expected if the associated shape features had independent histories of stabilizing selection in the two parental taxa. Combining two of these opposite effect QTLs could be expected to generate transgression (see e.g., Rieseberg et al., 1999). However, there were only a couple of transgressive individuals on this PC axis and it is unclear, which exact aspect of shape drives the association with the QTLs.

The detection of a marginally significant QTL for one of the behaviors in males (number of strikes) is surprising given our low sample size (*n* = 19), and should be interpreted with some caution. However, the pattern that males homozygous for CAL alleles had the highest values and males homozygous for TAE the lowest is consistent with the observation that CAL males had higher means in their number of strikes than TAE males (see Figure 4 and Figure S1).

In conclusion, we demonstrate a case where hybridization between two Lake Malawi cichlid species that are trophically unspecialized has not only resulted in transgression in morphology, but also in transgressive trait values in behaviors related to a feeding behavior that is not typical for either parental species but characterizes a whole clade of trophically specialized species in the Lake Malawi cichlid radiation. We find several QTLs for morphology, suggesting the existence of some loci with moderate to large effects. The genetic basis of transgression in this cross, however, is not yet resolved. One issue in our study was the relatively small sample sizes, which may make detection of QTLs difficult, especially those with small effect (see e.g., Slate, 2017), and will usually lead to overestimation of the effect of significant QTLs (Beavis, 1995). Furthermore, in addition to complementary gene action and overdominance, epistasis could also be involved in generating transgression (Rieseberg et al., 1999). The latter is still challenging to infer and would need more elaborate analyses (see e.g., Laurie, Wang, Carlini‐Garcia, & Zeng, 2014). Nevertheless, the functional morphology and/or behavior, which may underlie the effective and efficient use of a resource neither parental species has specialized on, is present in some of our F2 hybrids. If this occurred in the wild, in the situation where specialized sand sifters were not yet present, it may permit some hybrid genotypes to tap into niches not used by either parental taxon, as has also been demonstrated for natural hybrids in several plant taxa (Anton, R. Ward, & Cruzan, 2013; Donovan, Rosenthal, Sanchez‐Velenosi, Rieseberg, & Ludwig, 2010; Xing et al., 2014; Zhao et al., 2014) and for experimental hybrids in Lake Victoria cichlid fish (Selz & Seehausen, 2019). If hybrids have elevated fitness in some ecological conditions, but are less fit than the parentals in the latter's ecological conditions, this might enable ecological niche partitioning between a population of some hybrid genotypes and both parental species (Seehausen, 2004). If niche differentiation leads to habitat isolation, this could be one route to the evolution of reproductive isolation between a hybrid lineage and its progenitors (Buerkle, Morris, Asmussen, & Rieseberg, 2000; Mallet, 2007; Rieseberg et al., 1999). The alternative route to hybrid speciation would be if niche differentiation becomes genetically coupled to behavioral reproductive isolation (Selz, Thommen, et al., 2014). Interestingly, we see evidence for both in the hybrid cross we studied here: we see transgression and novelty in these hybrids in morphology (Selz, Lucek, et al., 2014; Stelkens et al., 2009; and the present study) and in a behavior linked to utilizing a resource not typical of either parent (present study), and also in traits that are relevant for reproductive behavior (assortative mate choice and transgressive male color in F1 hybrids in Selz, Thommen, et al., 2014). If both assortative mating and the potential to adapt to a novel food resource come together in hybrids—and indications for both exist in this experimental species cross—this might enable a new hybrid species to emerge relatively quickly. Together, these findings suggest that interspecific hybridization in cichlids can generate functional novelty that is known from other cichlid species to be adaptive in certain ecological contexts and could thereby possibly facilitate or have facilitated niche shifts, promoting speciation, and adaptive radiation.

## CONFLICT OF INTEREST

We declare we have no competing interests.

## AUTHOR CONTRIBUTIONS

**Anna F. Feller:** Conceptualization (supporting); Data curation (lead); Formal analysis (lead); Investigation (lead); Methodology (lead); Visualization (lead); Writing‐original draft (lead); Writing‐review & editing (lead). **Oliver M. Selz:** Conceptualization (equal); Formal analysis (supporting); Investigation (supporting); Methodology (supporting); Supervision (equal); Writing‐review & editing (equal). **Matthew D. McGee:** Conceptualization (supporting); Investigation (supporting); Methodology (supporting); Supervision (equal); Writing‐review & editing (supporting). **Joana I. Meier:** Formal analysis (supporting); Supervision (supporting); Writing‐review & editing (equal). **Salome Mwaiko:** Investigation (supporting); Resources (supporting); Writing‐review & editing (supporting). **Ole Seehausen:** Conceptualization (lead); Formal analysis (supporting); Funding acquisition (lead); Investigation (supporting); Methodology (supporting); Project administration (lead); Resources (lead); Supervision (lead); Visualization (supporting); Writing‐review & editing (equal).

## Supporting information

Appendix S1

## Data Availability

Raw read (fastq) files for all genotyped individuals are available on the NCBI short read archive (SRA) under accession number PRJNA612298. The filtered vcf file, linkage map, phenotype‐genotype table, the TPS files, and an overview of which individuals were used in which part of the analyses are available on the Dryad Digital Repository at https://doi.org/10.5061/dryad.280gb5mn7.

## References

[ece36471-bib-0001] Abbott, R. , Albach, D. , Ansell, S. , Arntzen, J. W. , Baird, S. J. E. , Bierne, N. , … Zinner, D. (2013). Hybridization and speciation. Journal of Evolutionary Biology, 26, 229–246. 10.1111/j.1420-9101.2012.02599.x 23323997

[ece36471-bib-0002] Adams, D. C. (1999). Methods for shape analysis of landmark data from articulated structures. Evolutionary Ecology Research, 1, 959–970.

[ece36471-bib-0003] Adams, D. C. , & Otárola‐Castillo, E. (2013). geomorph: An R package for the collection and analysis of geometric morphometric shape data. Methods in Ecology and Evolution, 4, 393–399.

[ece36471-bib-0004] Albertson, R. C. , & Kocher, T. D. (2005). Genetic architecture sets limits on transgressive segregation in hybrid cichlid fishes. Evolution, 59, 686–690. 10.1111/j.0014-3820.2005.tb01027.x 15856710

[ece36471-bib-0005] Anderson, E. , & Stebbins, G. L. L. (1954). Hybridization as an evolutionary stimulus. Evolution, 8, 378–388. 10.1111/j.1558-5646.1954.tb01504.x

[ece36471-bib-0006] Anton, K. A. , R. Ward, J. , & Cruzan, M. B. (2013). Pollinator‐mediated selection on floral morphology: Evidence for transgressive evolution in a derived hybrid lineage. Journal of Evolutionary Biology, 26, 660–673. 10.1111/jeb.12083 23331370

[ece36471-bib-0007] Arnegard, M. E. , McGee, M. D. , Matthews, B. , Marchinko, K. B. , Conte, G. L. , Kabir, S. , … Schluter, D. (2014). Genetics of ecological divergence during speciation. Nature, 511, 307–311. 10.1038/nature13301 24909991 PMC4149549

[ece36471-bib-0008] Baird, N. A. , Etter, P. D. , Atwood, T. S. , Currey, M. C. , Shiver, A. L. , Lewis, Z. A. , … Johnson, E. A. (2008). Rapid SNP discovery and genetic mapping using sequenced RAD markers. PLoS One, 3, e3376 10.1371/journal.pone.0003376 18852878 PMC2557064

[ece36471-bib-0009] Barrett, R. D. H. , & Schluter, D. (2008). Adaptation from standing genetic variation. Trends in Ecology & Evolution, 23, 38–44. 10.1016/j.tree.2007.09.008 18006185

[ece36471-bib-0010] Barrier, M. , Baldwin, B. G. , Robichaux, R. H. , & Purugganan, M. D. (1999). Interspecific hybrid ancestry of a plant adaptive radiation: Allopolyploidy of the Hawaiian silversword alliance (Asteraceae) inferred from floral homeotic gene duplications. Molecular Biology and Evolution, 16, 1105–1113. 10.1093/oxfordjournals.molbev.a026200 10474905

[ece36471-bib-0011] Beavis, W. D. (1995). The power and deceit of QTL experiments: Lessons from comparative QTL studies. In (Ed.), Proceedings of the 49th Annual Corn and Sorghum Industry Research Conference, ASTA, Washington (pp. 252–268).

[ece36471-bib-0012] Bell, M. A. , & Travis, M. P. (2005). Hybridization, transgressive segregation, genetic covariation, and adaptive radiation. Trends in Ecology & Evolution, 20, 358–361. 16701394 10.1016/j.tree.2005.04.021

[ece36471-bib-0013] Benjamini, Y. , & Hochberg, Y. (1995). Controlling the false discovery rate: A practical and powerful approach to multiple testing. Journal of the Royal Statistical Society. Series B (Methodological), 57, 289–300.

[ece36471-bib-0014] Bookstein, F. L. (1997). Shape and the Information in Medical Images: A decade of the Morphometric Synthesis. Computer Vision and Image Understanding Proc. 66(2), 97–118.

[ece36471-bib-0016] Broman, K. W. , Wu, H. , Sen, S. , & Churchill, G. A. (2003). R/qtl: QTL mapping in experimental crosses. Bioinformatics, 19, 889–890. 10.1093/bioinformatics/btg112 12724300

[ece36471-bib-0017] Buerkle, C. A. , Morris, R. J. , Asmussen, M. A. , & Rieseberg, L. H. (2000). The likelihood of homoploid hybrid speciation. Heredity, 84, 441 10.1046/j.1365-2540.2000.00680.x 10849068

[ece36471-bib-0018] Catchen, J. , Hohenlohe, P. A. , Bassham, S. , Amores, A. , & Cresko, W. A. (2013). Stacks: An analysis tool set for population genomics. Molecular Ecology, 22, 3124–3140. 10.1111/mec.12354 23701397 PMC3936987

[ece36471-bib-0019] Danecek, P. , Auton, A. , Abecasis, G. , Albers, C. A. , Banks, E. , DePristo, M. A. , … Durbin, R. (2011). The variant call format and VCFtools. Bioinformatics, 27, 2156–2158. 10.1093/bioinformatics/btr330 21653522 PMC3137218

[ece36471-bib-0020] DeMarais, B. D. , Dowling, T. E. , Douglas, M. E. , Minckley, W. L. , & Marsh, P. C. (1992). Origin of Gila seminuda (Teleostei: Cyprinidae) through introgressive hybridization: Implications for evolution and conservation. Proceedings of the National Academy of Sciences of the United States of America, 89, 2747–2751. 10.1073/pnas.89.7.2747 1557380 PMC48739

[ece36471-bib-0021] Donovan, L. A. , Rosenthal, D. R. , Sanchez‐Velenosi, M. , Rieseberg, L. H. , & Ludwig, F. (2010). Are hybrid species more fit than ancestral parent species in the current hybrid species habitats? Journal of Evolutionary Biology, 23, 805–816. 10.1111/j.1420-9101.2010.01950.x 20210826

[ece36471-bib-0022] Fay, M. P. , & Shaw, P. A. (2010). Exact and asymptotic weighted logrank tests for interval censored data: The interval R package. Journal of Statistical Software, 36, 1–34. 10.18637/jss.v036.i02 PMC4184046 25285054

[ece36471-bib-0023] Feulner, P. G. D. , Schwarzer, J. , Haesler, M. P. , Meier, J. I. , & Seehausen, O. (2018). A dense linkage map of lake victoria cichlids improved the pundamilia genome assembly and revealed a major QTL for sex‐determination. G3; Genes|Genomes|Genetics, 8, 2411–2420. 29760203 10.1534/g3.118.200207 PMC6027883

[ece36471-bib-0024] Fox, J. , & Weisberg, S. (2011). An R companion to applied regression. London, UK: SAGE Publications, Inc..

[ece36471-bib-0025] Fryer, G. , & Iles, T. D. (1972). The cichlid fishes of the great lakes of Africa: Their biology and evolution. Edinburgh, UK: Oliver & Boyd.

[ece36471-bib-0026] Grant, B. R. , & Grant, P. R. (1996). High survival of Darwin’ s Finch hybrids : Effects of beak morphology and diets. Ecology, 77, 500–509.

[ece36471-bib-0027] Grant, B. R. , & Grant, P. R. (2008). Fission and fusion of Darwin’s Finches populations. Philosophical Transactions of the Royal Society B: Biological Sciences, 363, 2821–2829. 10.1098/rstb.2008.0051 PMC2606742 18508750

[ece36471-bib-0028] Grant, P. R. , & Grant, B. R. (1992). Hybridization of Bird Species. Science, 256, 193–197. 10.1126/science.256.5054.193 17744718

[ece36471-bib-0029] Greenwood, P. H. (1974). Cichlid fishes of Lake Victoria, East Africa: The biology and evolution of a species flock. Bristol, UK: John Wright and Sons Ltd., Stonebridge Press.

[ece36471-bib-0030] Hedrick, P. W. (2013). Adaptive introgression in animals: Examples and comparison to new mutation and standing variation as sources of adaptive variation. Molecular Ecology, 22, 4606–4618. 10.1111/mec.12415 23906376

[ece36471-bib-0031] Holzman, R. , & Hulsey, C. D. (2017). Mechanical transgressive segregation and the rapid origin of trophic novelty. Scientific Reports, 7(40306), 1–15. 10.1038/srep40306 28079133 PMC5228120

[ece36471-bib-0032] Hudson, A. G. , Vonlanthen, P. , & Seehausen, O. (2011). Rapid parallel adaptive radiations from a single hybridogenic ancestral population. Proceedings of the Royal Society B‐Biological Sciences, 278, 58–66. 10.1098/rspb.2010.0925 PMC2992718 20685705

[ece36471-bib-0033] Joyce, D. A. , Lunt, D. H. , Genner, M. J. , Turner, G. F. , Bills, R. , & Seehausen, O. (2011). Repeated colonization and hybridization in Lake Malawi cichlids. Current Biology, 21, 526 10.1016/j.cub.2011.02.044 21300271

[ece36471-bib-0034] Kagawa, K. , & Takimoto, G. (2017). Hybridization can promote adaptive radiation by means of transgressive segregation. Ecology Letters, 21, 264–274. 10.1111/ele.12891 29243294

[ece36471-bib-0035] Kane, E. A. , & Higham, T. E. (2015). Complex systems are more than the sum of their parts: Using integration to understand performance, biomechanics, and diversity. Integrative and Comparative Biology, 55, 146–165. 10.1093/icb/icv033 25979469

[ece36471-bib-0036] Keller, I. , Wagner, C. E. , Greuter, L. , Mwaiko, S. , Selz, O. M. , Sivasundar, A. et al. (2013). Population genomic signatures of divergent adaptation, gene flow and hybrid speciation in the rapid radiation of Lake Victoria cichlid fishes. Molecular Ecology, 22, 2848–2863. 23121191 10.1111/mec.12083

[ece36471-bib-0037] Klingenberg, C. P. (2016). Size, shape, and form: Concepts of allometry in geometric morphometrics. Development Genes and Evolution, 226, 113–137. 27038023 10.1007/s00427-016-0539-2 PMC4896994

[ece36471-bib-0038] Kocher, T. D. (2004). Adaptive evolution and explosive speciation: The cichlid fish model. Nature Reviews Genetics, 5, 288–298. 10.1038/nrg1316 15131652

[ece36471-bib-0039] Konings, A. (2010). Malawi cichlids in their natural habitat. El Paso, TX: Cichlid Press.

[ece36471-bib-0040] Lamichhaney, S. , Berglund, J. , Almén, M. S. , Maqbool, K. , Grabherr, M. , Martinez‐Barrio, A. , … Andersson, L. (2015). Evolution of Darwin’s finches and their beaks revealed by genome sequencing. Nature, 518, 371–375. 10.1038/nature14181 25686609

[ece36471-bib-0041] Lamichhaney, S. , Han, F. , Webster, M. T. , Andersson, L. , Grant, B. R. , & Grant, P. R. (2017). Rapid hybrid speciation in Darwin’s finches. Science, 359, 224–228. 10.1126/science.aao4593 29170277

[ece36471-bib-0042] Lander, E. S. , & Botstein, S. (1989). Mapping mendelian factors underlying quantitative traits using RFLP linkage maps. Genetics, 121, 185–199. 2563713 10.1093/genetics/121.1.185 PMC1203601

[ece36471-bib-0043] Langmead, B. , & Salzberg, S. L. (2012). Fast gapped‐read alignment with Bowtie 2. Nature Methods, 9, 357–359. 10.1038/nmeth.1923 22388286 PMC3322381

[ece36471-bib-0044] Laurie, C. , Wang, S. , Carlini‐Garcia, L. A. , & Zeng, Z. B. (2014). Mapping epistatic quantitative trait loci. BMC Genetics, 15(112), 1–13. 10.1186/s12863-014-0112-9 25367219 PMC4226885

[ece36471-bib-0045] Lewontin, R. C. , & Birch, L. C. (1966). Hybridization as a source of variation for adaptation to new environments. Evolution, 20, 315–336. 10.1111/j.1558-5646.1966.tb03369.x 28562982

[ece36471-bib-0046] Lexer, C. , Welch, M. E. , Raymond, O. , & Rieseberg, L. H. (2003). The origin of ecological divergence in helianthus paradoxus (asteraceae): Selection on transgressive characters in a novel hybrid habitat. Evolution, 57, 1989–2000. 10.1111/j.0014-3820.2003.tb00379.x 14575321

[ece36471-bib-0047] Losos, J. B. (2010). Adaptive radiation, ecological opportunity, and evolutionary determinism. American Naturalist, 175, 623–639. 10.1086/652433 20412015

[ece36471-bib-0048] Malinsky, M. , Svardal, H. , Tyers, A. M. , Miska, E. A. , Genner, M. J. , Turner, G. F. , & Durbin, R. (2018). Whole genome sequences of Malawi cichlids reveal multiple radiations interconnected by gene flow. Nature Ecology & Evolution, 2, 1940–1955. 30455444 10.1038/s41559-018-0717-x PMC6443041

[ece36471-bib-0049] Mallet, J. (2007). Hybrid speciation. Nature, 446, 279–283. 10.1038/nature05706 17361174

[ece36471-bib-0050] Marques, D. A. , Lucek, K. , Meier, J. I. , Mwaiko, S. , Wagner, C. E. , Excoffier, L. , & Seehausen, O. (2016). Genomics of rapid incipient speciation in sympatric threespine stickleback. PLoS Genetics, 12, e1005887 10.1371/journal.pgen.1005887 26925837 PMC4771382

[ece36471-bib-0051] Marques, D. A. , Meier, J. I. , & Seehausen, O. (2019). A combinatorial view on speciation and adaptive radiation. Trends in Ecology & Evolution, 34, 531–544. 10.1016/j.tree.2019.02.008 30885412

[ece36471-bib-0052] Martin, C. H. , & Richards, E. J. (2019). The paradox behind the pattern of rapid adaptive radiation: How can the speciation process sustain itself through an early burst? Annual Review of Ecology Evolution and Systematics, 50, 569–593. 10.1146/annurev-ecolsys-110617-062443 PMC9555815 36237480

[ece36471-bib-0053] McGee, M. D. , Borstein, S. R. , Meier, J. I. , Marques, D. A. , Mwaiko, S. , Taabu, A. , … & Seehausen, O. et al. (in press). The ecological and genomic basis of explosive adaptive radiation. Nature. 10.1038/s41586-020-2652-7 32848251

[ece36471-bib-0054] McGee, M. D. , Schluter, D. , & Wainwright, P. C. (2013). Functional basis of ecological divergence in sympatric stickleback. BMC Evolutionary Biology, 13, 1–10. 10.1186/1471-2148-13-277 24380474 PMC3890603

[ece36471-bib-0055] McKaye, K. R. , Stauffer, J. R. J. , Turner, G. F. , Konings, A. , & Sato, T. (2001). Fishes, as well as birds, build bowers. Journal of Aquariculture and Aquatic Sciences, 9, 121–133.

[ece36471-bib-0056] McKenna, A. , Hanna, M. , Banks, E. , Sivachenko, A. , Cibulskis, K. , Kernytsky, A. , … DePristo, M. A. (2010). The Genome Analysis Toolkit: A MapReduce framework for analyzing next‐generation DNA sequencing data. Genome Research, 20, 1297–1303. 10.1101/gr.107524.110 20644199 PMC2928508

[ece36471-bib-0057] Meier, J. I. , Marques, D. A. , Mwaiko, S. , Wagner, C. E. , Excoffier, L. , & Seehausen, O. (2017). Ancient hybridization fuels rapid cichlid fish adaptive radiations. Nature Communications, 8(14363), 1–11. 10.1038/ncomms14363 PMC5309898 28186104

[ece36471-bib-0058] Meier, J. I. , Stelkens, R. B. , Joyce, D. A. , Mwaiko, S. , Phiri, N. , Schliewen, U. K. , Selz, O. M. , Wagner, C. E. , Katongo, C. , & Seehausen, O. (2019). The coincidence of ecological opportunity with hybridization explains rapid adaptive radiation in Lake Mweru cichlid fishes. Nature Communications, 10(5391), 1–11. 10.1038/s41467-019-13278-z PMC6890737 31796733

[ece36471-bib-0059] Meyer, B. S. , Matschiner, M. , & Salzburger, W. (2017). Disentangling incomplete lineage sorting and introgression to refine species‐tree estimates for Lake Tanganyika cichlid fishes. Systematic Biology, 66, 531–550. 27539485 10.1093/sysbio/syw069

[ece36471-bib-0060] Parnell, N. F. , Hulsey, C. D. , & Streelman, J. T. (2008). Hybridization produces novelty when the mapping of form to function is many to one. BMC Evolutionary Biology, 8, 122 10.1186/1471-2148-8-122 18442407 PMC2386449

[ece36471-bib-0061] Parnell, N. F. , Hulsey, C. D. , & Streelman, J. T. (2012). The genetic basis of a complex functional system. Evolution, 66, 3352–3366. 10.1111/j.1558-5646.2012.01688.x 23106702 PMC3490443

[ece36471-bib-0062] Parsons, K. J. , Cooper, W. J. , & Albertson, R. C. (2011). Modularity of the oral jaws is linked to repeated changes in the craniofacial shape of African cichlids. International Journal of Evolutionary Biology, 2011, 1–10. 10.4061/2011/641501 PMC3119590 21716745

[ece36471-bib-0063] Parsons, K. J. , Son, Y. H. , & Craig Albertson, R. (2011). Hybridization promotes evolvability in African cichlids: Connections between transgressive segregation and phenotypic integration. Evolutionary Biology, 38, 306–315. 10.1007/s11692-011-9126-7

[ece36471-bib-0064] Pereira, R. J. , Barreto, F. S. , & Burton, R. S. (2014). Ecological novelty by hybridization: Experimental evidence for increased thermal tolerance by transgressive segregation in *Tigriopus californicus* . Evolution, 68, 204–215. 24372605 10.1111/evo.12254

[ece36471-bib-0065] Pitcher, T. J. (1982). Heuristic definitions of fish shoaling behaviour. Animal Behavior, 31, 611–613. 10.1016/S0003-3472(83)80087-6

[ece36471-bib-0066] R Development Core Team . (2015). R: A language and environment for statistical computing. Vienna, Austria: R Foundation for Statistical Computing.

[ece36471-bib-0067] Rasband, W. (2015). ImageJ [Software].

[ece36471-bib-0068] Rieseberg, L. H. , Archer, M. A. , & Wayne, R. K. (1999). Transgressive segregation, adaptation and speciation. Heredity, 83, 363–372. 10583537 10.1038/sj.hdy.6886170

[ece36471-bib-0069] Rieseberg, L. H. , Widmer, A. , Arntz, A. M. , & Burke, B. (2003). The genetic architecture necessary for transgressive segregation is common in both natural and domesticated populations. Philosophical Transactions of the Royal Society of London. Series B: Biological Sciences, 358, 1141–1147. 10.1098/rstb.2003.1283 12831480 PMC1693210

[ece36471-bib-0070] Rohlf, F. J. (2010). TPSdig version 2.16. Ecology & Evolution. Stony Brook, NY: SUNY.

[ece36471-bib-0071] Salzburger, W. (2018). Understanding explosive diversification through cichlid fish genomics. Nature Reviews Genetics, 19, 705–717. 10.1038/s41576-018-0043-9 30111830

[ece36471-bib-0072] Sambrook, J. , & Russell, D. W. (2001). Molecular cloning: A laboratory manual (3rd ed.). Cold Spring Harbor, NY: Cold Spring Harbor Laboratory Press.

[ece36471-bib-0073] Schliewen, U. K. , & Klee, B. (2004). Reticulate sympatric speciation in Cameroonian crater lake cichlids. Frontiers in Zoology, 1, 5. 15679917 10.1186/1742-9994-1-5 PMC544937

[ece36471-bib-0074] Schluter, D. (2000). The ecology of adaptive radiation.Oxford Series in Ecology and Evolution Oxford, UK: Oxford University Press.

[ece36471-bib-0075] Schluter, D. (2001). Ecology and the origin of species. Trends in Ecology & Evolution, 16, 372–380. 10.1016/S0169-5347(01)02198-X 11403870

[ece36471-bib-0076] Seehausen, O. (1996). Lake Victoria Rock Cichlids – taxonomy, ecology, and distribution. Zevenjuizen, the Netherlands: Verduijn Cichlids.

[ece36471-bib-0077] Seehausen, O. (2004). Hybridization and adaptive radiation. Trends in Ecology & Evolution, 19, 198–207. 10.1016/j.tree.2004.01.003 16701254

[ece36471-bib-0078] Seehausen, O. (2006). African cichlid fish: A model system in adaptive radiation research. Proceedings of the Royal Society B: Biological Sciences, 273, 1987–1998. 10.1098/rspb.2006.3539 PMC1635482 16846905

[ece36471-bib-0079] Selz, O. M. , Lucek, K. , Young, K. A. , & Seehausen, O. (2014). Relaxed trait covariance in interspecific cichlid hybrids predicts morphological diversity in adaptive radiations. Journal of Evolutionary Biology, 27, 11–24. 10.1111/jeb.12283 24330234

[ece36471-bib-0080] Selz, O. M. , & Seehausen, O. (2019). Interspecific hybridization can generate functional novelty in cichlid fish. Proceedings of the Royal Society B: Biological Sciences, 286, 20191621 10.1098/rspb.2019.1621 PMC6834035 31640510

[ece36471-bib-0081] Selz, O. M. , Thommen, R. , Maan, M. E. , & Seehausen, O. (2014). Behavioural isolation may facilitate homoploid hybrid speciation in cichlid fish. Journal of Evolutionary Biology, 27, 275–289. 10.1111/jeb.12287 24372872

[ece36471-bib-0082] Slate, J. (2017). Robust inference of genetic architecture in mapping studies. Molecular Ecology, 26, 1453–1455. 10.1111/mec.14052 28299864

[ece36471-bib-0083] Slatkin, M. , & Lande, R. (1994). Segregation variance after hybridization of isolated populations. Genetical Research, 64, 51–56. 7958831 10.1017/s0016672300032547

[ece36471-bib-0084] Stebbins, G. L. (1959). The role of hybridization in evolution. Proceedings of the American Philosophical Society, 103, 231–251.

[ece36471-bib-0015] Simakov Oleg , Ng Alvin Y. , Lim Zhi Wei , Bezault Etienne , Turner‐Maier Jason , Johnson Jeremy , … & Di Palma F. (2014). The genomic substrate for adaptive radiation in African cichlid fish. Nature, 513, (7518), 375–381. 10.1038/nature13726. 25186727 PMC4353498

[ece36471-bib-0085] Stelkens, R. B. , Schmid, C. , Selz, O. , & Seehausen, O. (2009). Phenotypic novelty in experimental hybrids is predicted by the genetic distance between species of cichlid fish. BMC Evolutionary Biology, 9, 283 10.1186/1471-2148-9-283 19961584 PMC2796671

[ece36471-bib-0086] Stelkens, R. B. , & Seehausen, O. (2009). Phenotypic divergence but not genetic distance predicts assortative mating among species of a cichlid fish radiation. Journal of Evolutionary Biology, 22, 1679–1694. 10.1111/j.1420-9101.2009.01777.x 19549141

[ece36471-bib-0087] Svardal, H. , Quah, F. X. , Malinsky, M. , Ngatunga, B. P. , Miska, E. A. , Salzburger, W. , … Durbin, R. (2020). Ancestral hybridization facilitated species diversification in the lake Malawi cichlid fish adaptive radiation. Molecular Biology and Evolution, 37, 1100–1113. 10.1093/molbev/msz294 31821500 PMC7086168

[ece36471-bib-0088] van Ooijen, J. W. (2006). JoinMap^®^ 4, software for the calculation of genetic linkage maps in experimental populations, Wageningen, NL: Kyazama.

[ece36471-bib-0089] Verhoeven, K. J. F. , Simonsen, K. L. , & McIntyre, L. (2005). Implementing false discovery rate control: Increasing your power. Oikos, 108, 643–647. 10.1111/j.0030-1299.2005.13727.x

[ece36471-bib-0090] Xing, F. , Mao, J. F. , Meng, J. , Dai, J. , Zhao, W. , Liu, H. et al. (2014). Needle morphological evidence of the homoploid hybrid origin of *Pinus densata* based on analysis of artificial hybrids and the putative parents, *Pinus tabuliformis* and *Pinus yunnanensis* . Ecology and Evolution, 4, 1890–1902. 24963383 10.1002/ece3.1062 PMC4063482

[ece36471-bib-0091] York, R. A. , Patil, C. , Hulsey, C. D. , Streelman, J. T. , & Fernald, R. D. (2015). Evolution of bower building in Lake Malawi cichlid fish: phylogeny, morphology, and behavior. Frontiers in Ecology and Evolution, 3(18), 1–13. 10.3389/fevo.2015.00018

[ece36471-bib-0092] Young, K. A. , Snoeks, J. , & Seehausen, O. (2009). Morphological diversity and the roles of contingency, chance and determinism in African cichlid radiations. PLoS One, 4, e4740. 19270732 10.1371/journal.pone.0004740 PMC2648897

[ece36471-bib-0093] Zhao, W. , Meng, J. , Wang, B. , Zhang, L. , Xu, Y. , Zeng, Q. Y. et al (2014). Weak crossability barrier but strong juvenile selection supports ecological speciation of the hybrid pine *Pinus densata* on the Tibetan Plateau. Evolution, 68, 3120–3133. 25065387 10.1111/evo.12496 PMC4278550

