# Supplementary material for: Rapid generation of ecologically relevant behavioral novelty in experimental cichlid hybrids
Source: Ecol Evol. 2020 Jun 16;10(14):7445–62. doi: 10.1002/ece3.6471 (PMC7391563; doi:10.1002/ece3.6471)
Supplement: Supplementary file 1 — Appendix S1 [file ECE3-10-7445-s001.docx]

**Appendix**

**List of the 21 sand sifting species from Lake Malawi** that were used for geometric morphometric analyses (one specimen of each species, with one exception (*Taeniolethrinops laticeps* from two populations)):

*Ctenopharynx intermedius*

*Ctenopharynx nitidus*

*Fossorochromis rostratus*

*Lethrinops altus*

*Lethrinops argenteus*

*Lethrinops aurita*

*Lethrinops gossei*

*Lethrinops leptodon*

*Lethrinops lethrinus*

*Lethrinops lunaris*

*Lethrinops macracanthus*

*Lethrinops macrochir*

*Otopharynx tetrastigma*

*Tramitichromis brevis*

*Taeniolethrinops cyrtonotus*

*Taeniolethrinops furcicauda*

*Taeniolethrinops laticeps*

*Taeniolethrinops laticeps2*

*Tramitichromis liturus*

*Taeniolethrinops praeorbitalis*

*Tramitichromis trilineatus*

| **Table S1** Linear trait values for F2 hybrids (n=161) and parental species (CAL n= 26, TAE n=24). Mean (mean), standard deviation (sd), maximum (max) and minimum (min) trait values for each class are shown as well as transgression thresholds (tg thresholds; see Methods) and the proportion of transgressive F2 individuals. Bold values indicate values ranging above (max) or below (min) the thresholds calculated from the parental species. The top part of the table includes all individuals of a given class, the subsequent parts are only males of a given class, or females, respectively. | | | | | | |
| --- | --- | --- | --- | --- | --- | --- |
|  | EyL_all | EpD_all | LJL_all | ML_all | MA_all | HW_all |
| *A. calliptera* |  |  |  |  |  |  |
| mean | -0.022 | -0.011 | 0.051 | 0.049 | 41.871 | 0.012 |
| sd | 0.028 | 0.039 | 0.041 | 0.032 | 4.333 | 0.035 |
| max | 0.043 | 0.093 | 0.126 | 0.111 | 50.068 | 0.085 |
| min | -0.075 | -0.090 | -0.039 | -0.016 | 29.846 | -0.049 |
| *P. taeniolatus* |  |  |  |  |  |  |
| mean | 0.003 | 0.037 | -0.073 | -0.070 | 35.625 | -0.033 |
| sd | 0.021 | 0.028 | 0.035 | 0.035 | 3.538 | 0.018 |
| max | 0.050 | 0.085 | 0.003 | -0.009 | 41.310 | 0.003 |
| min | -0.039 | -0.017 | -0.149 | -0.140 | 28.977 | -0.076 |
| high tg threshold | 0.044 | 0.092 | 0.133 | 0.114 | 50.537 | 0.083 |
| low tg threshold | -0.078 | -0.089 | -0.144 | -0.141 | 28.548 | -0.070 |
| *F2 hybrids* |  |  |  |  |  |  |
| mean | 0.003 | -0.004 | 0.003 | 0.002 | 36.991 | 0.003 |
| sd | 0.030 | 0.053 | 0.049 | 0.039 | 5.354 | 0.028 |
| max | **0.088** | **0.139** | 0.132 | 0.105 | **54.905** | **0.088** |
| min | -0.078 | **-0.178** | -0.124 | -0.122 | **20.295** | **-0.078** |
| prop tg high | 0.087 | 0.019 |  |  | 0.012 | 0.006 |
| prop tg low |  | 0.056 |  |  | 0.050 | 0.006 |

|  | EyL_M | EpD_M | LJL_M | ML_M | MA_M | HW_M |
| --- | --- | --- | --- | --- | --- | --- |
| *A. calliptera* |  |  |  |  |  |  |
| mean | -0.029 | 0.006 | 0.026 | 0.041 | 43.955 | 0.005 |
| sd | 0.023 | 0.028 | 0.044 | 0.037 | 4.109 | 0.025 |
| max | -0.002 | 0.068 | 0.088 | 0.105 | 50.068 | 0.036 |
| min | -0.075 | -0.018 | -0.039 | -0.016 | 37.908 | -0.028 |
| *P. taeniolatus* |  |  |  |  |  |  |
| mean | 0.009 | 0.042 | -0.066 | -0.051 | 35.075 | -0.036 |
| sd | 0.021 | 0.030 | 0.021 | 0.020 | 3.343 | 0.023 |
| max | 0.050 | 0.085 | -0.025 | -0.020 | 41.120 | 0.003 |
| min | -0.026 | -0.001 | -0.091 | -0.090 | 30.990 | -0.076 |
| high tg threshold | 0.050 | 0.102 | 0.114 | 0.115 | 52.173 | 0.054 |
| low tg threshold | -0.076 | -0.051 | -0.108 | -0.091 | 28.390 | -0.082 |
| *F2 hybrids* |  |  |  |  |  |  |
| mean | -0.009 | -0.002 | -0.004 | 0.002 | 34.371 | 0.000 |
| sd | 0.026 | 0.071 | 0.046 | 0.036 | 5.397 | 0.026 |
| max | **0.058** | **0.139** | 0.075 | 0.072 | 47.832 | **0.057** |
| min | **-0.078** | **-0.178** | -0.104 | **-0.100** | **22.069** | -0.078 |
| prop tg high | 0.020 | 0.039 |  |  |  | 0.039 |
| prop tg low | 0.020 | 0.176 |  | 0.020 | 0.118 |  |

|  | EyL_F | EpD_F | LJL_F | ML_F | MA_F | HW_F |
| --- | --- | --- | --- | --- | --- | --- |
| *A. calliptera* |  |  |  |  |  |  |
| mean | -0.019 | -0.018 | 0.062 | 0.053 | 40.944 | 0.015 |
| sd | 0.030 | 0.042 | 0.035 | 0.030 | 4.208 | 0.039 |
| max | 0.043 | 0.093 | 0.126 | 0.111 | 48.159 | 0.085 |
| min | -0.075 | -0.090 | -0.003 | 0.010 | 29.846 | -0.049 |
| *P. taeniolatus* |  |  |  |  |  |  |
| mean | 0.000 | 0.034 | -0.077 | -0.081 | 35.954 | -0.032 |
| sd | 0.020 | 0.027 | 0.042 | 0.039 | 3.724 | 0.015 |
| max | 0.043 | 0.070 | 0.003 | -0.009 | 41.310 | -0.001 |
| min | -0.039 | -0.017 | -0.149 | -0.140 | 28.977 | -0.049 |
| high tg threshold | 0.041 | 0.087 | 0.133 | 0.113 | 49.360 | 0.094 |
| low tg threshold | -0.079 | -0.102 | -0.161 | -0.158 | 28.505 | -0.062 |
| *F2 hybrids* |  |  |  |  |  |  |
| mean | 0.011 | -0.005 | 0.009 | 0.005 | 38.163 | 0.004 |
| sd | 0.029 | 0.043 | 0.047 | 0.039 | 4.903 | 0.030 |
| max | **0.088** | **0.100** | 0.132 | 0.105 | **54.905** | 0.088 |
| min | -0.054 | -0.092 | -0.124 | -0.122 | **20.295** | -0.062 |
| prop tg high | 0.137 | 0.010 |  |  | 0.020 |  |
| prop tg low |  |  |  |  | 0.020 |  |

| **Table S2** Trait loadings of PCA on linear traits | | | | | | |
| --- | --- | --- | --- | --- | --- | --- |
|  | PC1 | PC2 | PC3 | PC4 | PC5 | PC6 |
| EpD | -0.2921123 | -0.3251506134 | 0.60710282 | -0.44940555 | 0.4866553 | -0.03968547 |
| EyL | 0.2872742 | 0.4677250305 | 0.06324927 | -0.77444820 | -0.3069651 | 0.02662182 |
| LJL | 0.6268270 | -0.0005996981 | 0.22596467 | 0.15513248 | 0.1795698 | -0.70690598 |
| ML | 0.6278722 | -0.0915811033 | 0.13655805 | 0.09704739 | 0.2916979 | 0.69587025 |
| MA | 0.1371058 | -0.4324427123 | -0.72216129 | -0.39875779 | 0.3165696 | -0.11599268 |
| HW | 0.1618958 | -0.6929041903 | 0.19026462 | -0.07597754 | -0.6718554 | 0.01762293 |

| **Table S3.** Models testing for association of morphology with maximum observed (log10) efficiency in F2 hybrid males (n=23) | | | | | | | | | | | |
| --- | --- | --- | --- | --- | --- | --- | --- | --- | --- | --- | --- |
| model  (shape PC axes) | Estimate | Std. Error | t-value | Pr(>\|t\|) |  | model  (linear traits) | Estimate | Std. Error | t-value | Pr(>\|t\|) |  |
| efficiency ~ PC1 | -3.76 | 7.06 | -0.53 | 0.60 |  | efficiency ~ EpD | 1.16 | 2.49 | 0.47 | 0.65 |  |
| efficiency ~ PC2 | -0.73 | 8.09 | -0.09 | 0.93 |  | efficiency ~ EyL | 10.40 | 6.84 | 1.52 | 0.14 |  |
| efficiency ~ PC3 | 2.30 | 10.75 | 0.21 | 0.83 |  | efficiency ~ LJL | 2.81 | 4.52 | 0.62 | 0.54 |  |
| efficiency ~ PC4 | 14.39 | 9.98 | 1.44 | 0.16 |  | efficiency ~ ML | -1.31 | 5.68 | -0.23 | 0.82 |  |
| efficiency ~ PC5 | 7.54 | 9.66 | 0.78 | 0.44 |  | efficiency ~ MA | 0.03 | 0.04 | 0.77 | 0.45 |  |
| efficiency ~ PC6 | -12.50 | 15.15 | -0.83 | 0.42 |  | efficiency ~ HW | -5.52 | 5.81 | -0.95 | 0.35 |  |
| efficiency ~ PC1+PC2+PC3+PC4+PC5+PC6 | | | |  |  | efficiency ~ EpD+EyL+LJL+ML+MA+HW | | |  |  |  |
| PC1 | -6.62 | 7.08 | -0.94 | 0.36 |  | EpD | 3.15 | 2.52 | 1.25 | 0.23 |  |
| PC2 | 3.30 | 8.80 | 0.38 | 0.71 |  | EyL | 10.51 | 8.62 | 1.22 | 0.24 |  |
| PC3 | 5.78 | 11.96 | 0.48 | 0.64 |  | LJL | 15.85 | 9.14 | 1.73 | 0.10 |  |
| PC4 | 21.53 | 10.71 | 2.01 | 0.06 | . | ML | -22.74 | 12.36 | -1.84 | 0.08 | . |
| PC5 | 15.81 | 10.25 | 1.54 | 0.14 |  | MA | 0.03 | 0.04 | 0.80 | 0.44 |  |
| PC6 | -38.96 | 18.61 | -2.09 | 0.05 | . | HW | -2.00 | 6.62 | -0.30 | 0.77 |  |
| Residual standard error: 0.7105 on 16 degrees of freedom | | | | | | Residual standard error: 0.7104 on 16 degrees of freedom | | | | | |
| Multiple R-squared: 0.3188, Adjusted R-squared: 0.06332 | | | | | | Multiple R-squared: 0.319, Adjusted R-squared: 0.06363 | | | | | |
| F-statistic: 1.248 on 6 and 16 DF, p-value: 0.3343 | | | | | | F-statistic: 1.249 on 6 and 16 DF, p-value: 0.3338 | | | | | |
| AIC: 57.20243 |  |  |  |  |  | AIC: 57.19470 |  |  |  |  |  |
| efficiency ~ PC1+PC3+PC4+PC5+PC6 | | |  |  |  | efficiency ~ EpD+EyL+LJL+ML+MA | | |  |  |  |
| PC1 | -7.10 | 6.79 | -1.05 | 0.31 |  | EpD | 3.14 | 2.45 | 1.28 | 0.22 |  |
| PC3 | 3.84 | 10.51 | 0.37 | 0.72 |  | EyL | 11.24 | 8.05 | 1.40 | 0.18 |  |
| PC4 | 21.18 | 10.40 | 2.04 | 0.06 | . | LJL | 16.24 | 8.80 | 1.84 | 0.08 | . |
| PC5 | 15.78 | 9.99 | 1.58 | 0.13 |  | ML | -24.23 | 11.02 | -2.20 | 0.04 | * |
| PC6 | -36.83 | 17.27 | -2.13 | 0.05 | * | MA | 0.03 | 0.04 | 0.79 | 0.44 |  |
| Residual standard error: 0.6923 on 17 degrees of freedom | | | | | | Residual standard error: 0.6911 on 17 degrees of freedom | | | | | |
| Multiple R-squared: 0.3128, Adjusted R-squared: 0.1107 | | | | | | Multiple R-squared: 0.3151, Adjusted R-squared: 0.1137 | | | | | |
| F-statistic: 1.547 on 5 and 17 DF, p-value: 0.2279 | | | | | | F-statistic: 1.564 on 5 and 17 DF, p-value: 0.2231 | | | | | |
| AIC: 55.40407 |  |  |  |  |  | AIC: 55.3251 |  |  |  |  |  |
| efficiency ~ PC1+PC4+PC5+PC6 | | |  |  |  | efficiency ~ EpD+EyL+LJL+ML | | |  |  |  |
| PC1 | -7.18 | 6.62 | -1.09 | 0.29 |  | EpD | 2.85 | 2.40 | 1.19 | 0.25 |  |
| PC4 | 21.41 | 10.13 | 2.12 | 0.05 | * | EyL | 13.52 | 7.44 | 1.82 | 0.09 | . |
| PC5 | 15.88 | 9.74 | 1.63 | 0.12 |  | LJL | 14.31 | 8.37 | 1.71 | 0.10 |  |
| PC6 | -35.27 | 16.33 | -2.16 | 0.04 | * | ML | -22.75 | 10.74 | -2.12 | 0.05 | * |
| Residual standard error: 0.6755 on 18 degrees of freedom | | | | | | Residual standard error: 0.6839 on 18 degrees of freedom | | | | | |
| Multiple R-squared: 0.3074, Adjusted R-squared: 0.1535 | | | | | | Multiple R-squared: 0.29, Adjusted R-squared: 0.1322 | | | | | |
| F-statistic: 1.997 on 4 and 18 DF, p-value: 0.1382 | | | | | | F-statistic: 1.838 on 4 and 18 DF, p-value: 0.1656 | | | | | |
| AIC: 53.58411 |  |  |  |  |  | AIC: 54.15535 |  |  |  |  |  |
| **efficiency ~ PC4+PC5+PC6** | |  |  |  |  | **efficiency ~ EyL+LJL+ML** | |  |  |  |  |
| PC4 | 21.15 | 10.17 | 2.08 | 0.05 | . | EyL | 12.30 | 7.45 | 1.65 | 0.12 |  |
| PC5 | 13.50 | 9.53 | 1.42 | 0.17 |  | LJL | 12.51 | 8.32 | 1.50 | 0.15 |  |
| PC6 | -31.58 | 16.04 | -1.97 | 0.06 | . | ML | -19.00 | 10.38 | -1.83 | 0.08 | . |
| Residual standard error: 0.6786 on 19 degrees of freedom | | | | | | Residual standard error: 0.6913 on 19 degrees of freedom | | | | | |
| Multiple R-squared: 0.2621, Adjusted R-squared: 0.1456 | | | | | | Multiple R-squared: 0.2343, Adjusted R-squared: 0.1134 | | | | | |
| F-statistic: 2.249 on 3 and 19 DF, p-value: 0.1156 | | | | | | F-statistic: 1.938 on 3 and 19 DF, p-value: 0.1577 | | | | | |
| **AIC: 53.04077** |  |  |  |  |  | **AIC: 53.89132** |  |  |  |  |  |
| efficiency ~ PC4+PC6 | |  |  |  |  | efficiency ~ EyL+ML | |  |  |  |  |
| PC4 | 20.12 | 10.40 | 1.94 | 0.07 | . | EyL | 13.79 | 7.61 | 1.81 | 0.09 | . |
| PC6 | -23.25 | 15.29 | -1.52 | 0.14 |  | ML | -6.09 | 6.01 | -1.01 | 0.32 |  |
| Residual standard error: 0.6954 on 20 degrees of freedom | | | | | | Residual standard error: 0.7565 on 21 degrees of freedom | | | | | |
| Multiple R-squared: 0.1842, Adjusted R-squared: 0.1026 | | | | | | Multiple R-squared: 0.1432, Adjusted R-squared: 0.05756 | | | | | |
| F-statistic: 2.258 on 2 and 20 DF, p-value: 0.1305 | | | | | | F-statistic: 1.672 on 2 and 20 DF, p-value: 0.2131 | | | | | |
| AIC: 53.34805 |  |  |  |  |  | AIC: 54.47558 |  |  |  |  |  |
| Signif. codes: 0 ‘***’ 0.001 ‘**’ 0.01 ‘*’ 0.05 ‘.’ 0.1 ‘ ’ 1; p-values here are not corrected for multiple comparisons. The drop1 function was used to sequentially drop terms from the multiple linear models. Best model based on AIC in bold. | | | | | | | | | | | |


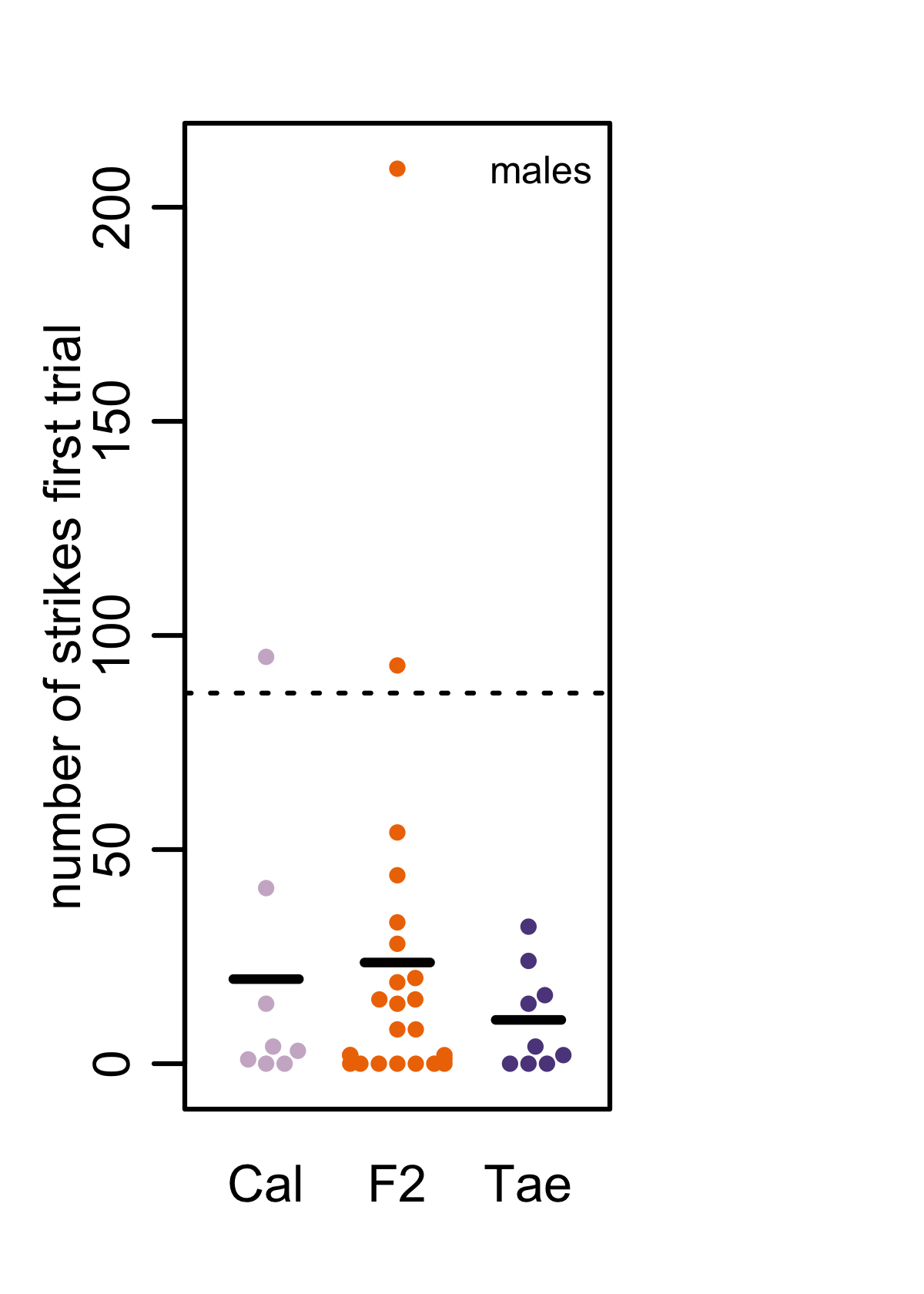

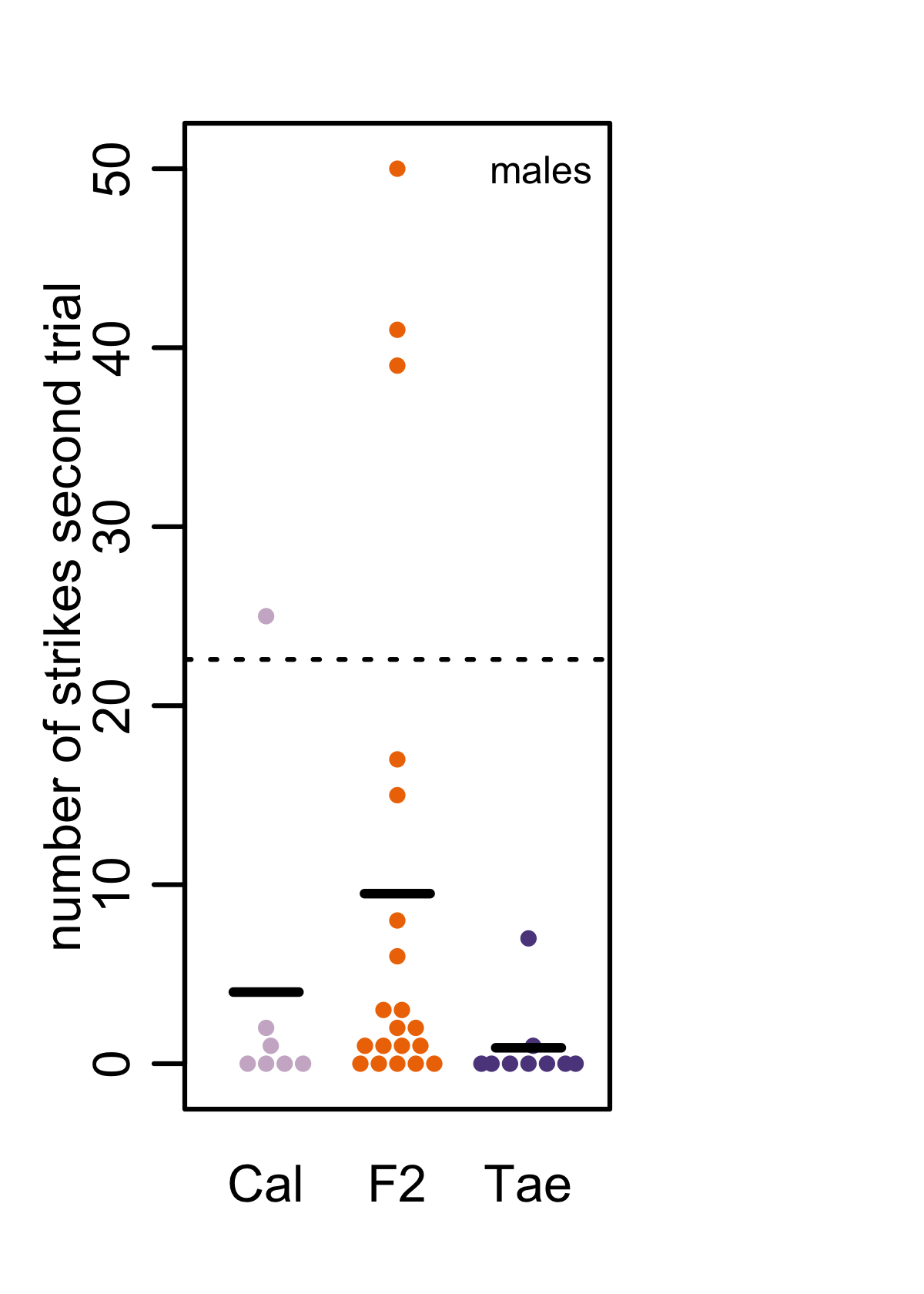

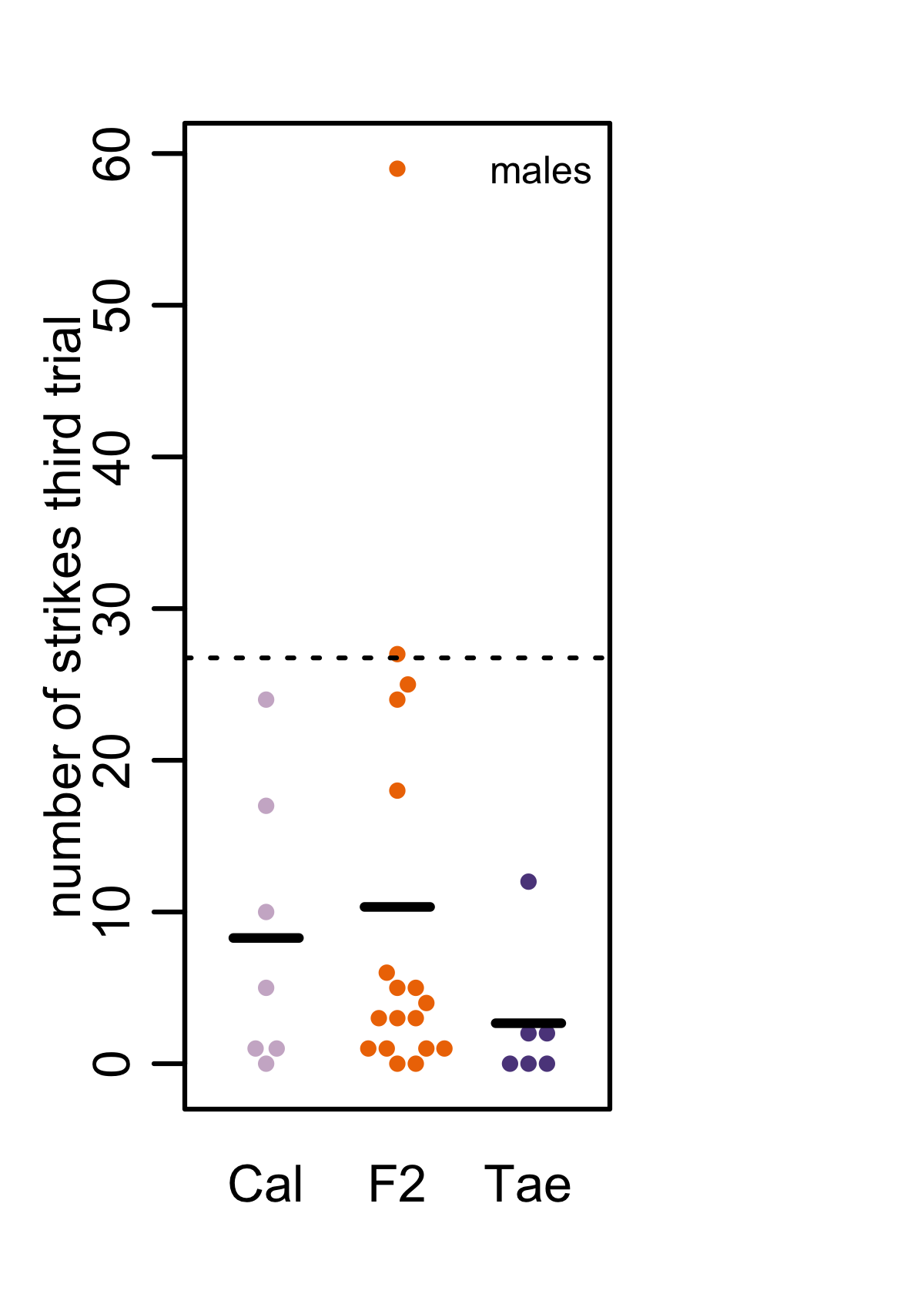

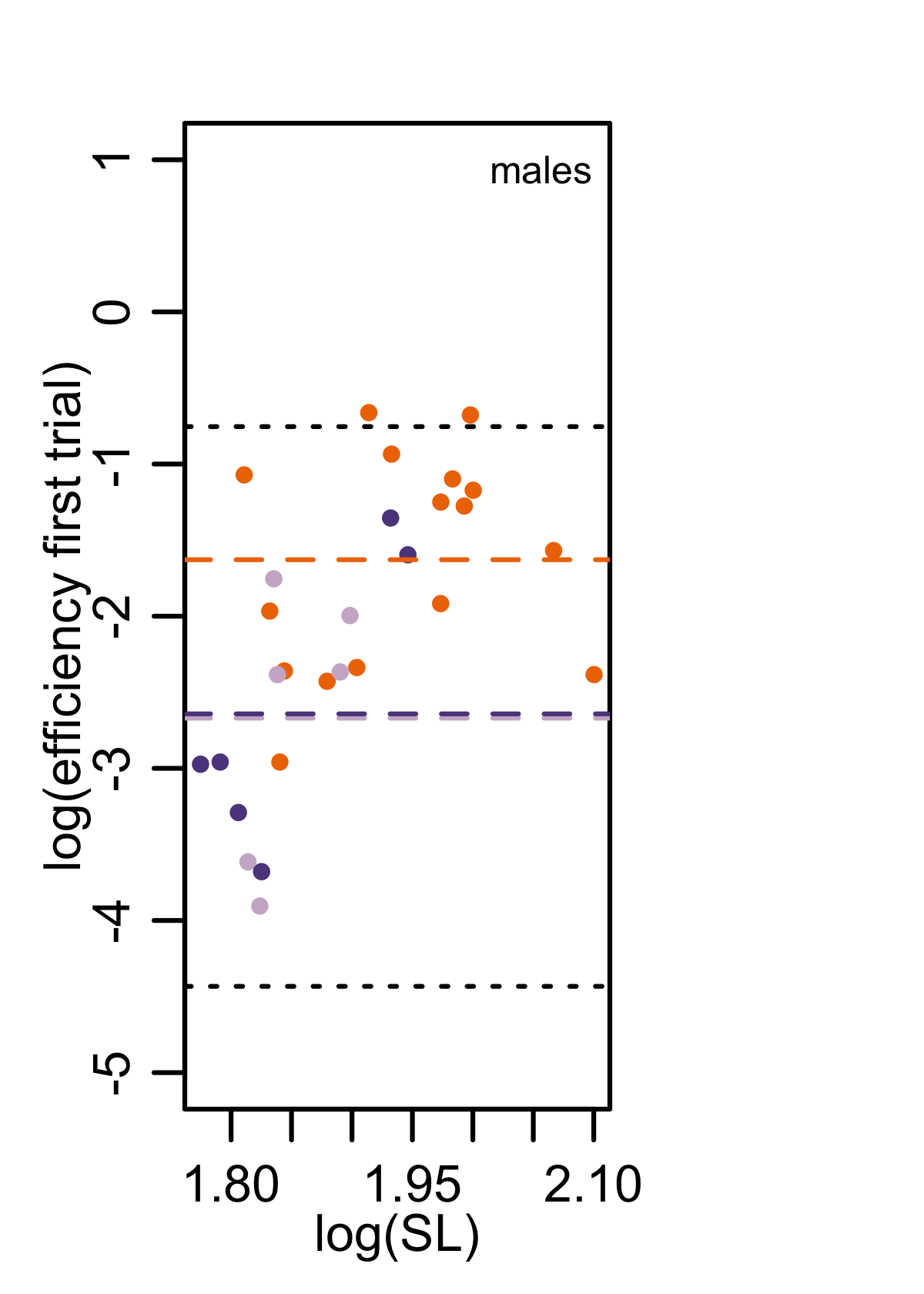

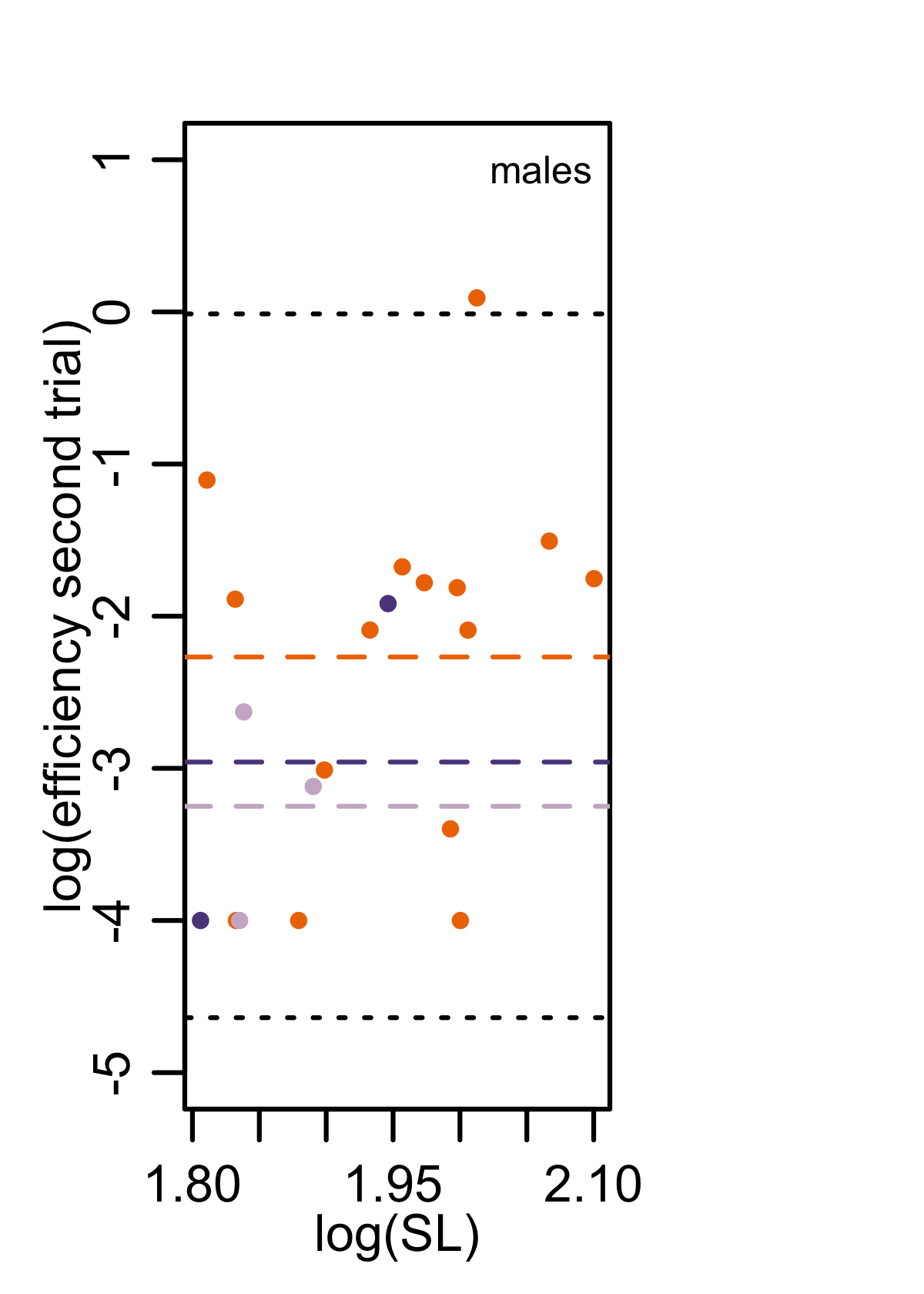

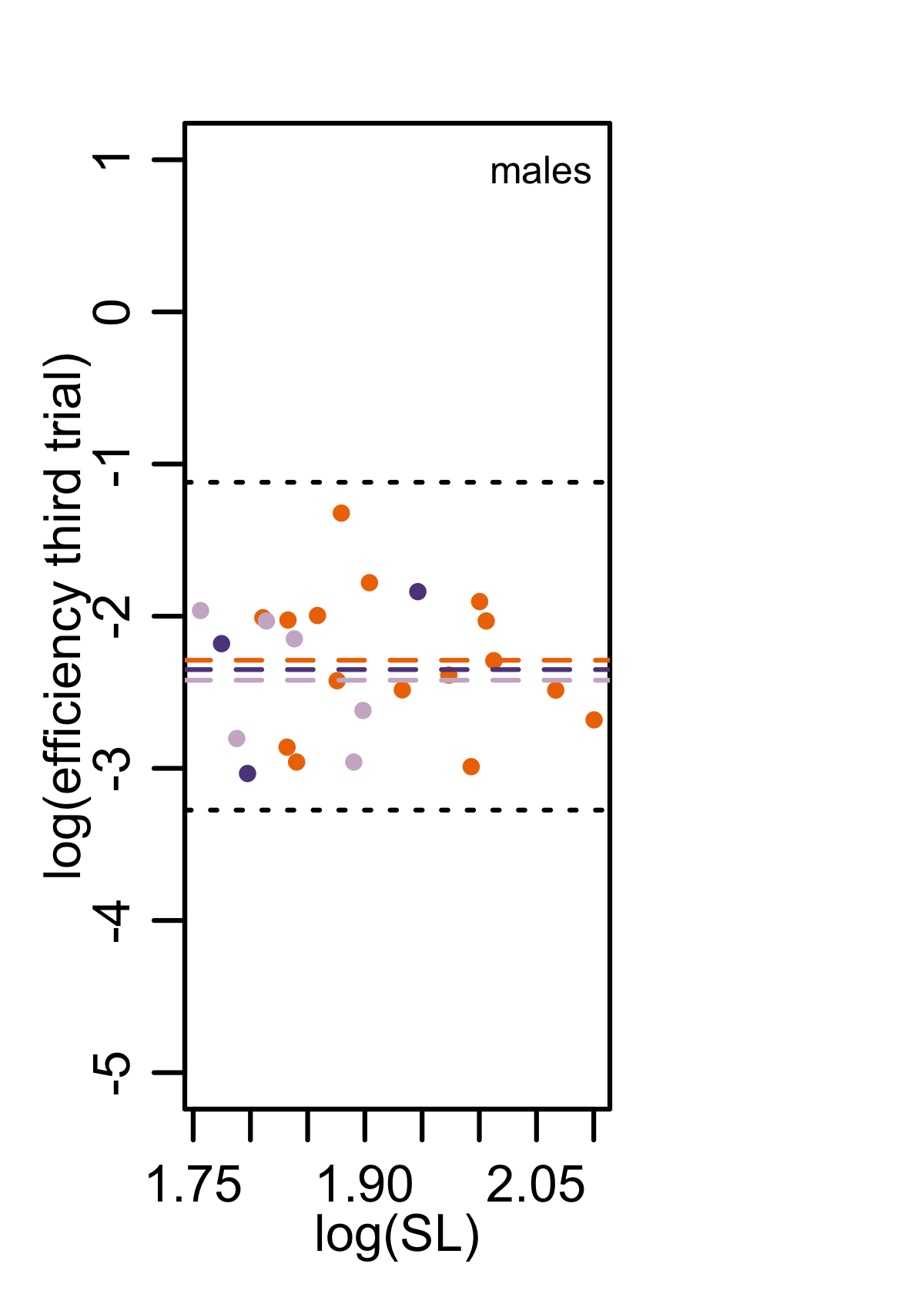

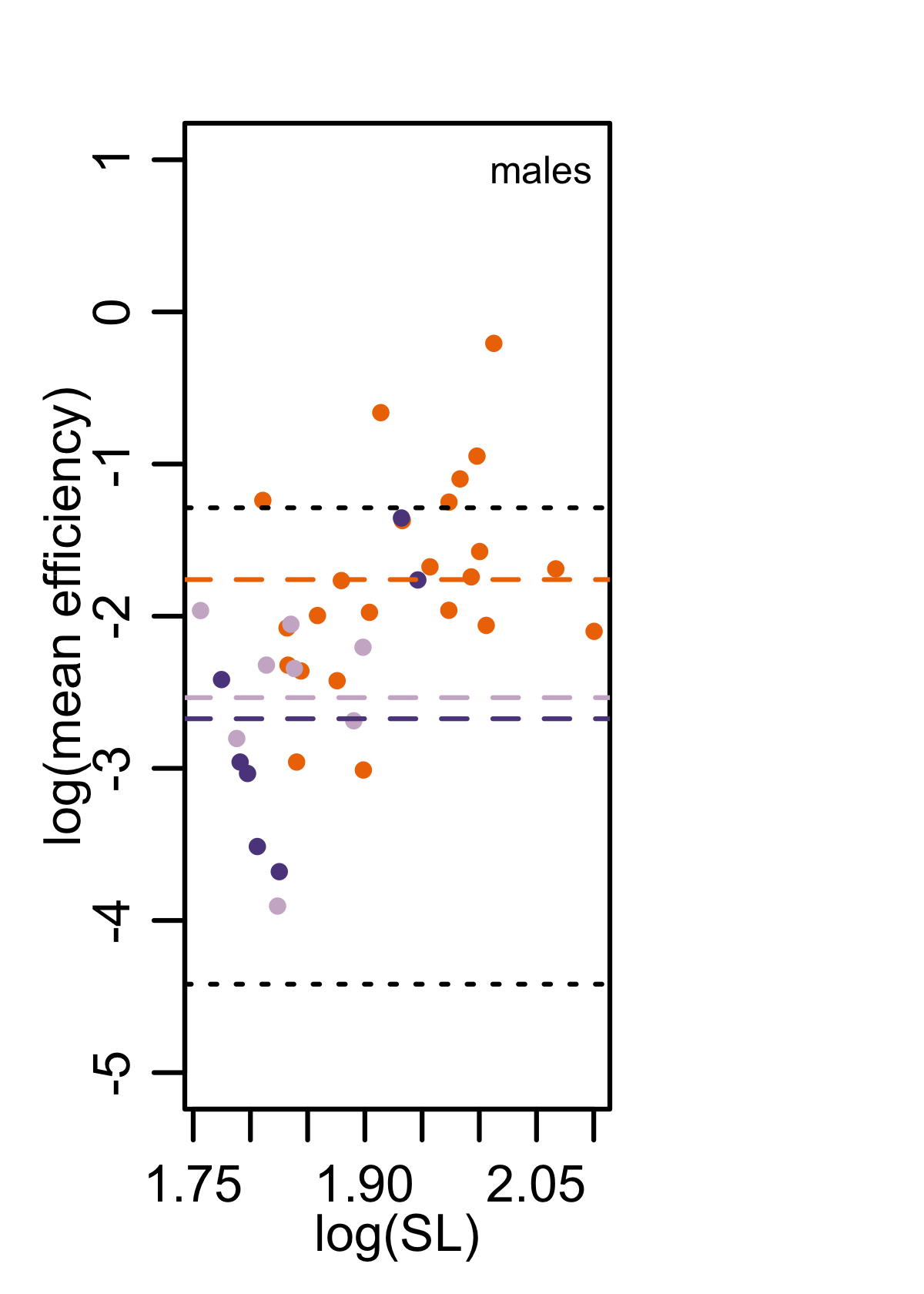

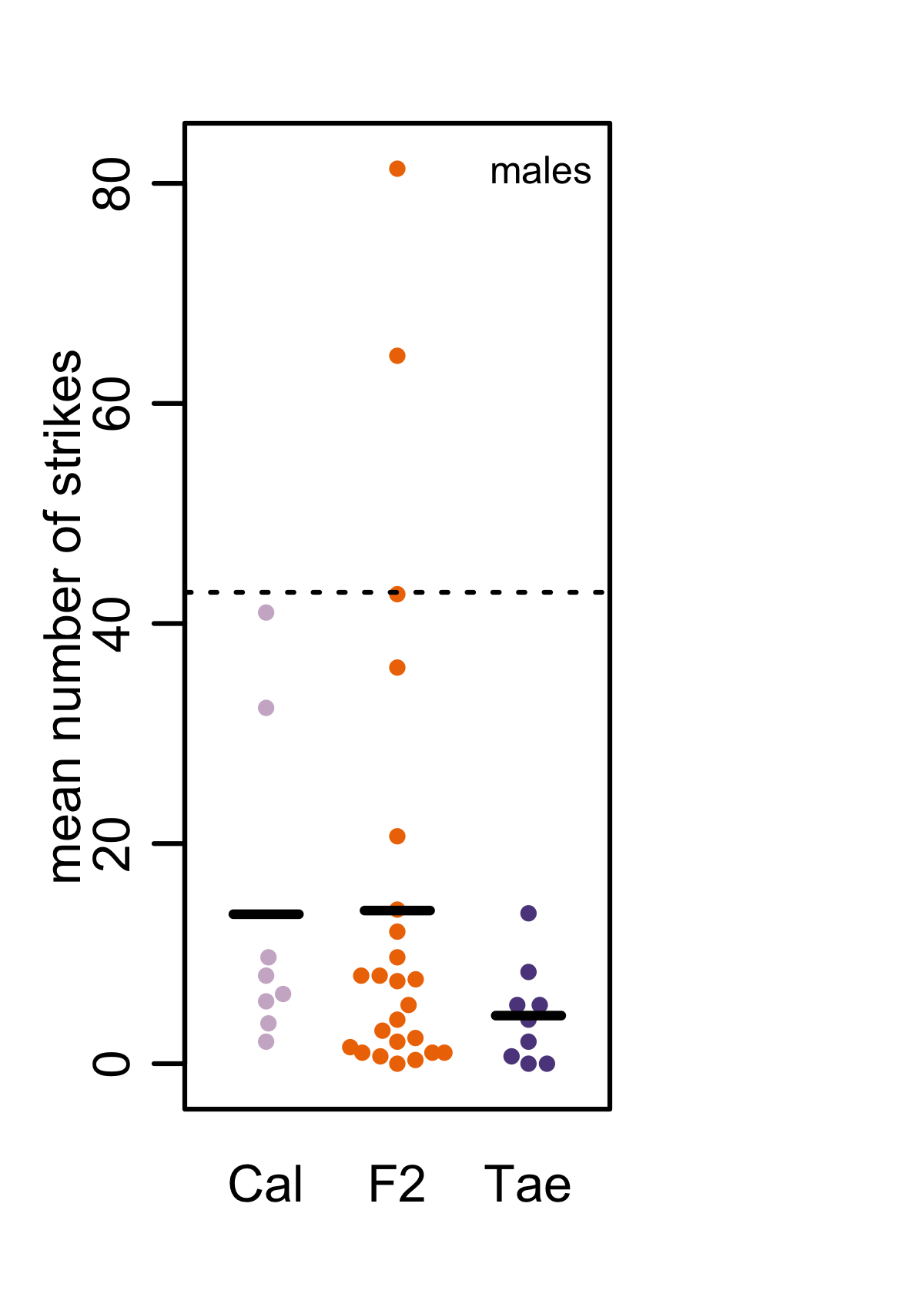


**Figure S1.** Per trial strikes and efficiency and means over all three trials in male sand sifting behavioural trials. The black dotted lines in all three plots indicate transgression thresholds given by the highest/lowest parental species mean +/- 2 standard deviations. The black bars in the top plot row indicate the mean in each class. The dashed lines with different colours in the bottom plot row indicate the mean in each class.


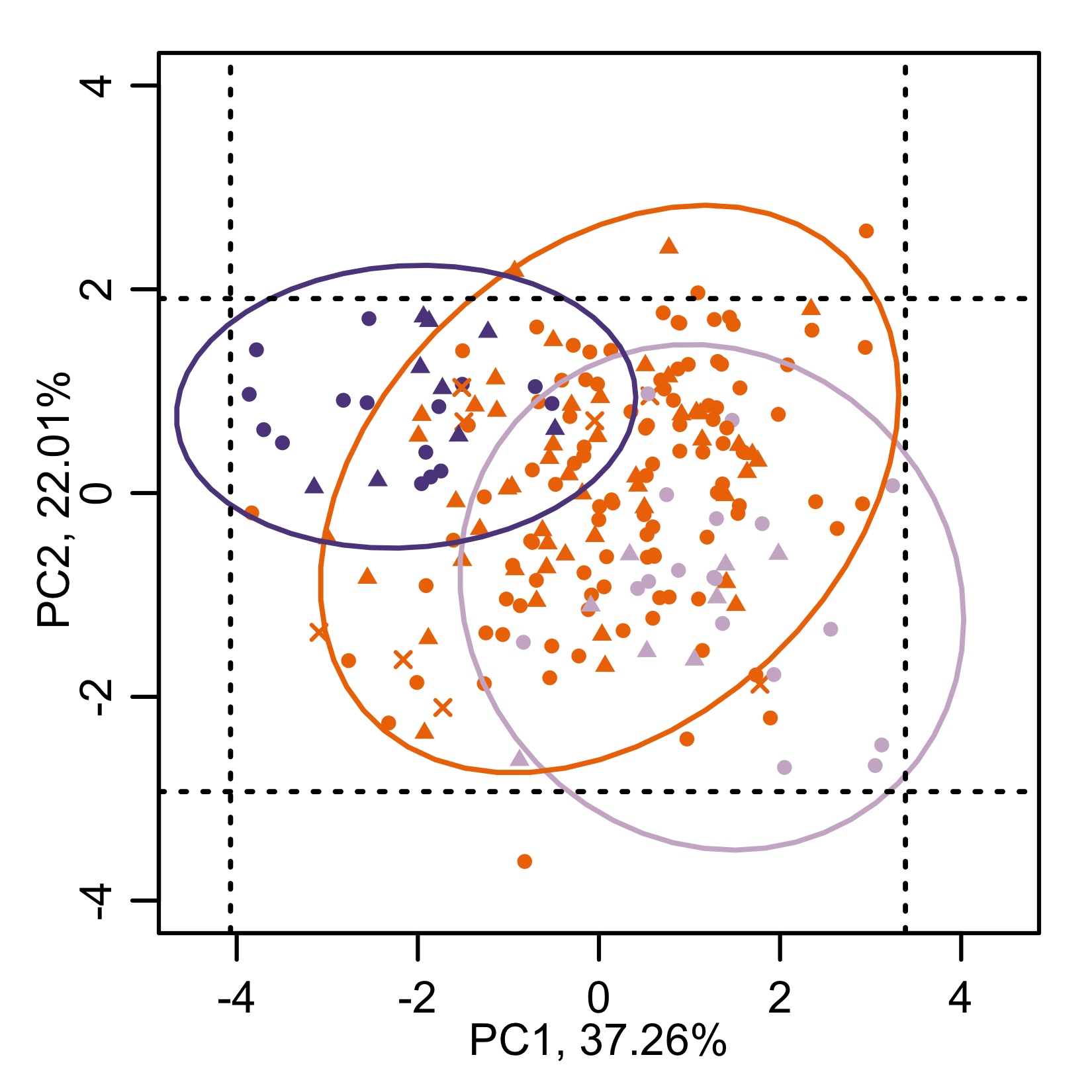

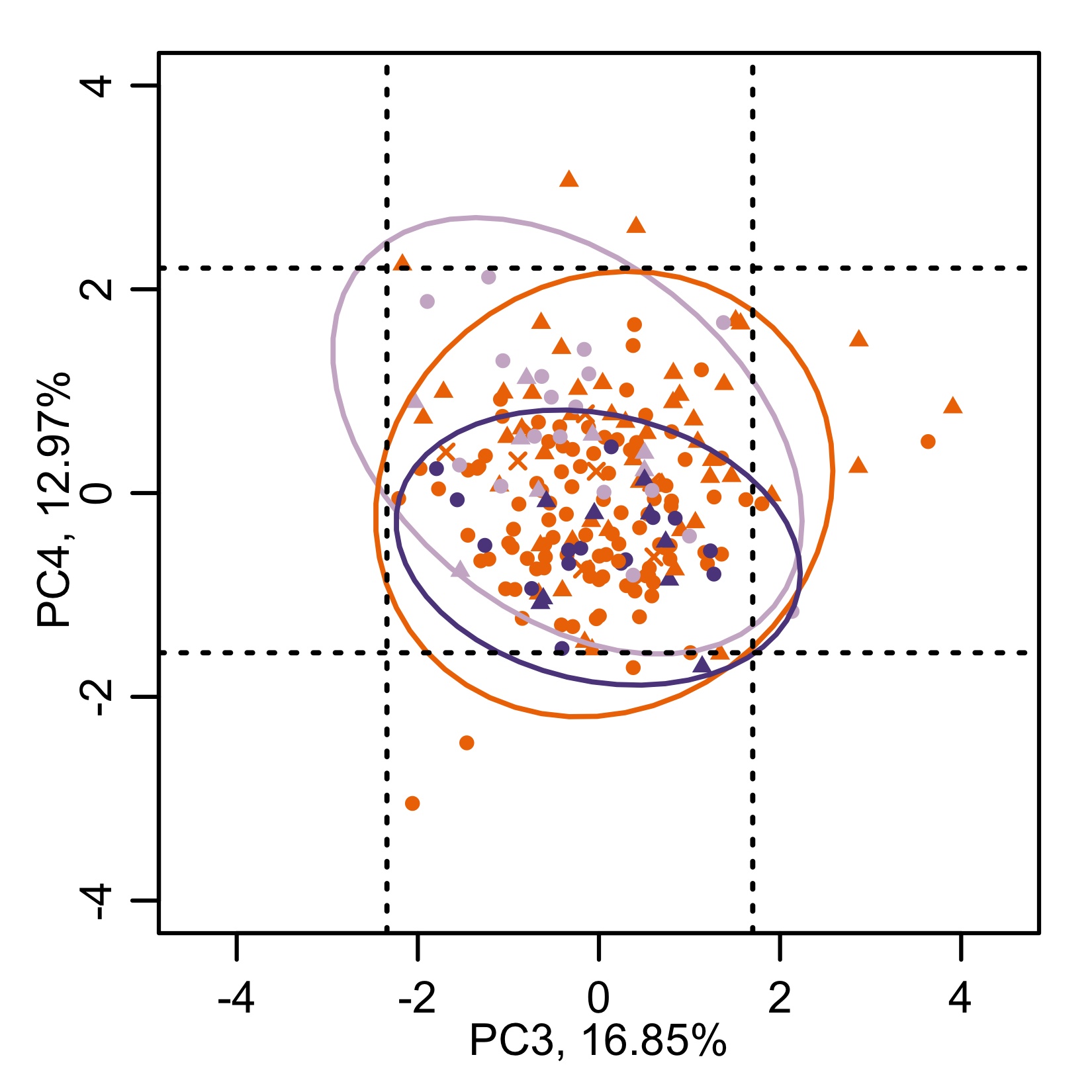

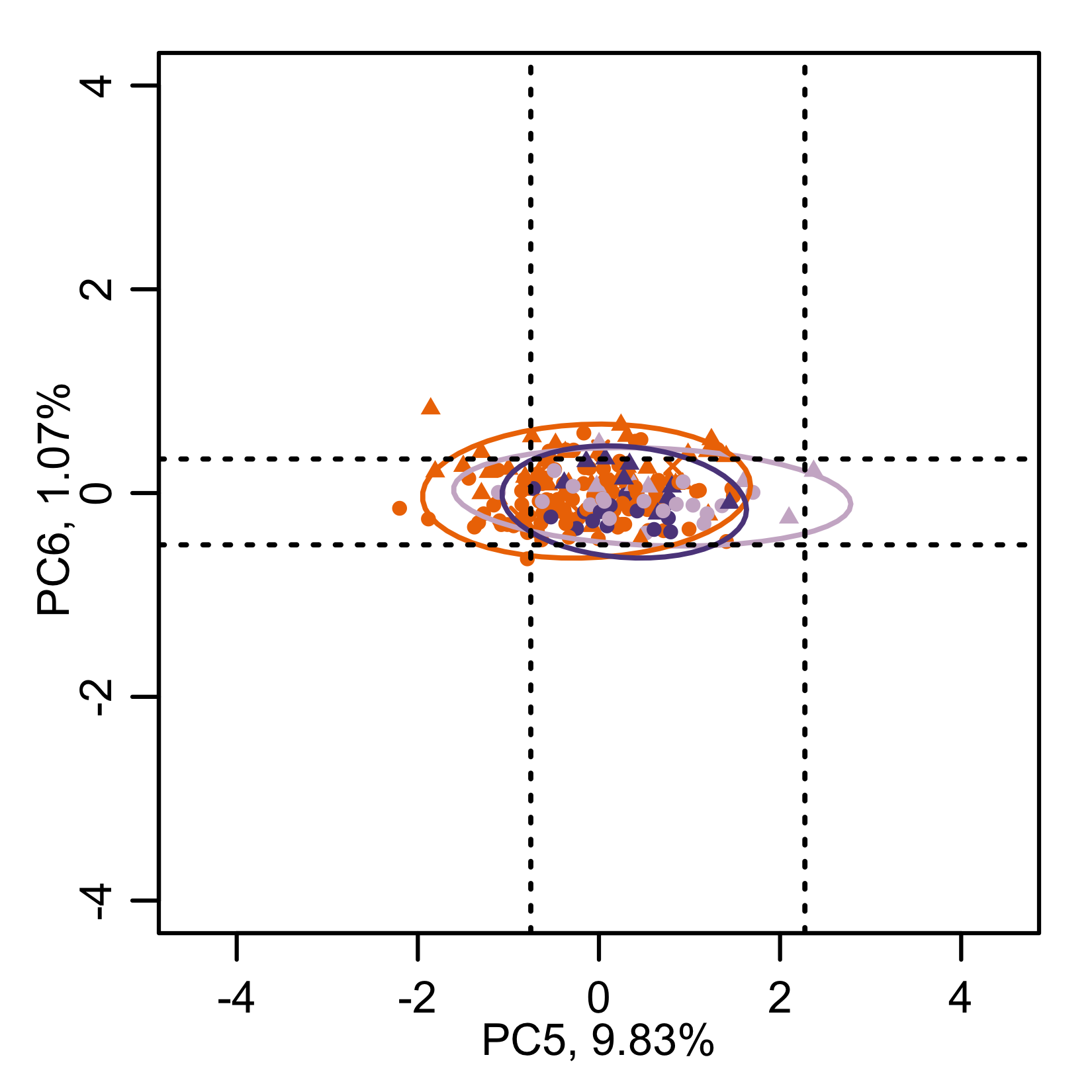

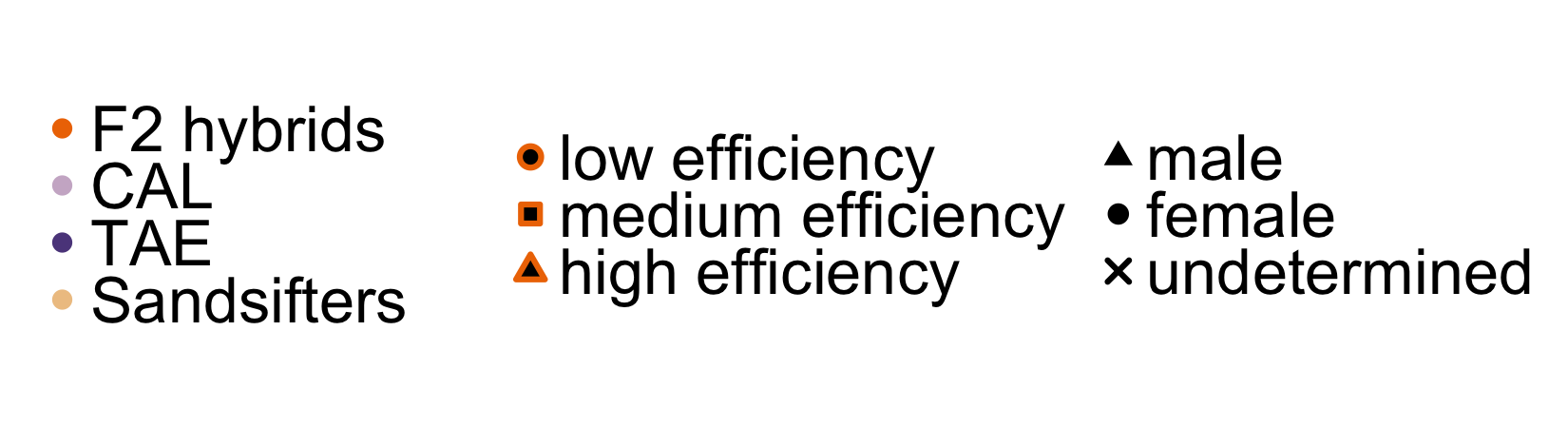

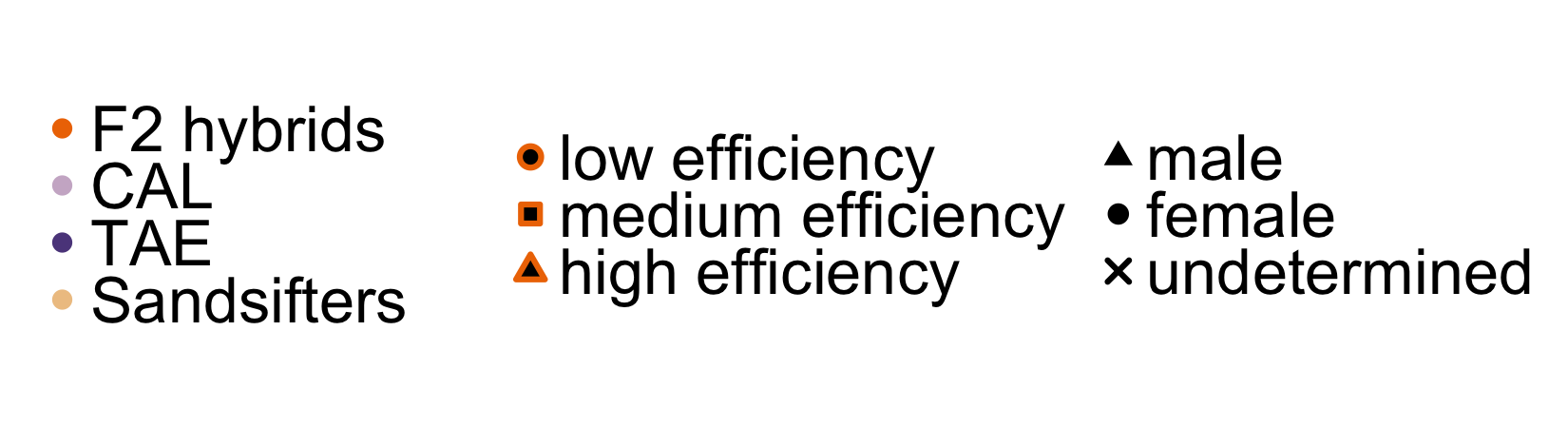


**(b)**

**(a)**

**(c)**

**Figure S2.** Principal component analyses (PCA) of linear traits. **(a)** PC1 vs PC2, **(b)** PC3 vs PC4, **(c)** PC5 vs PC6. The black dotted lines indicate transgression thresholds on a given axis given by the highest/lowest parental species mean value +/- 2 standard deviations. Ellipses represent 95% confidence intervals.


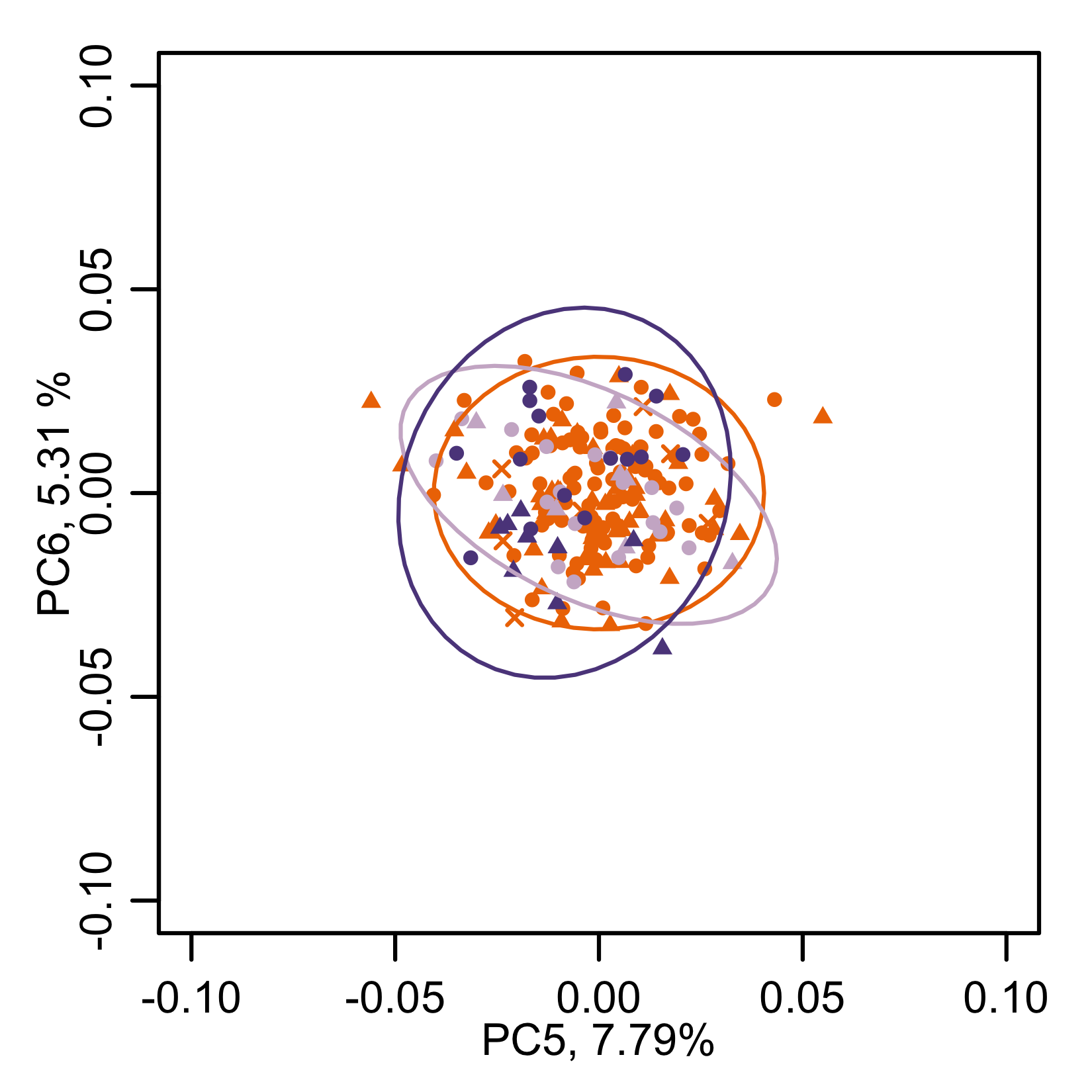

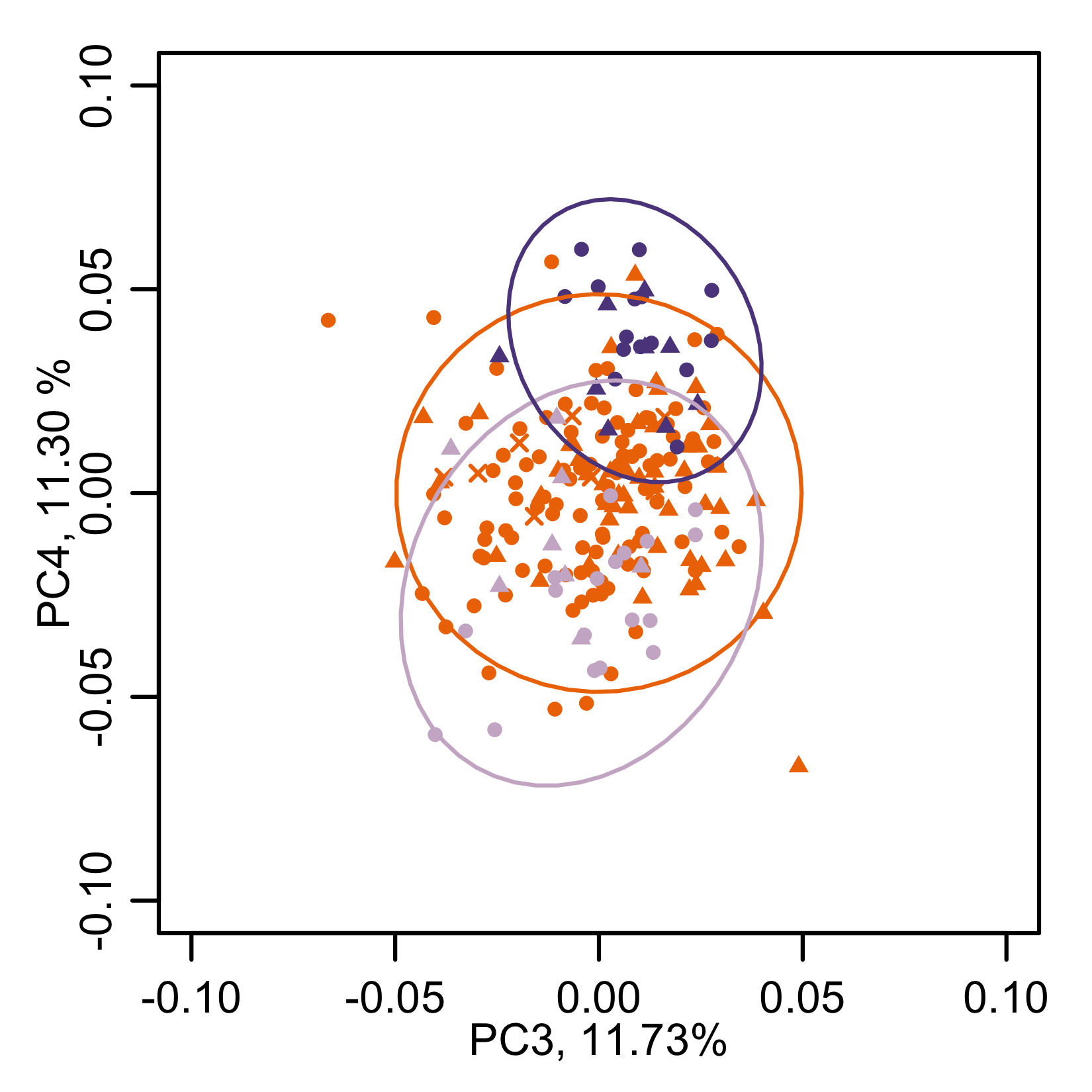

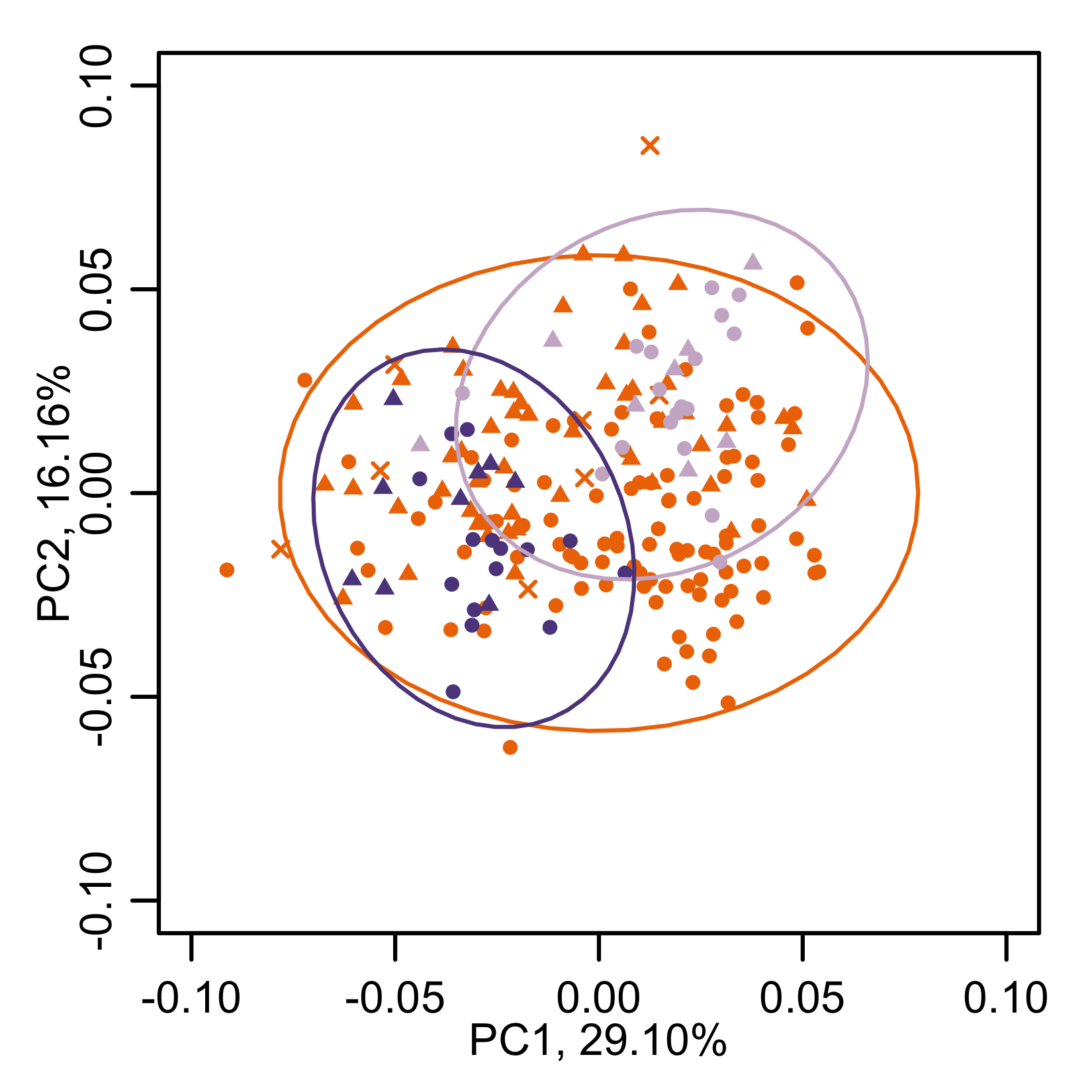

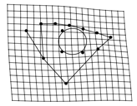

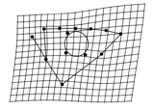

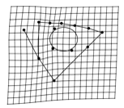

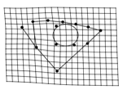

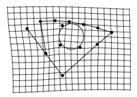

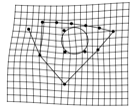

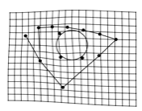

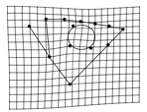

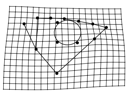

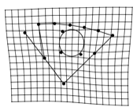

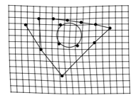

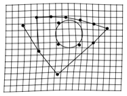

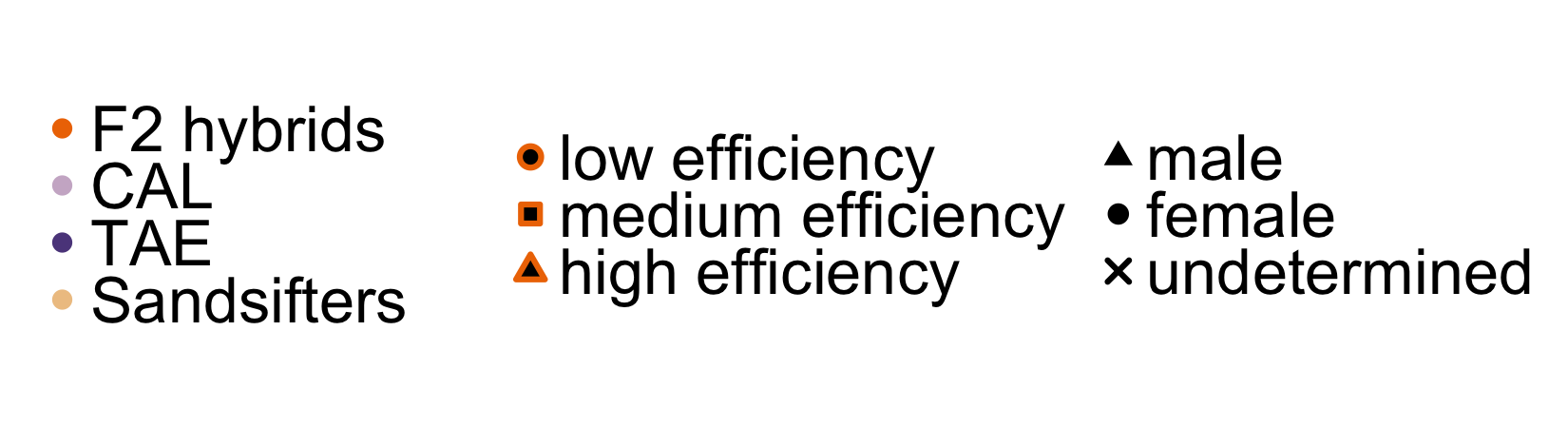

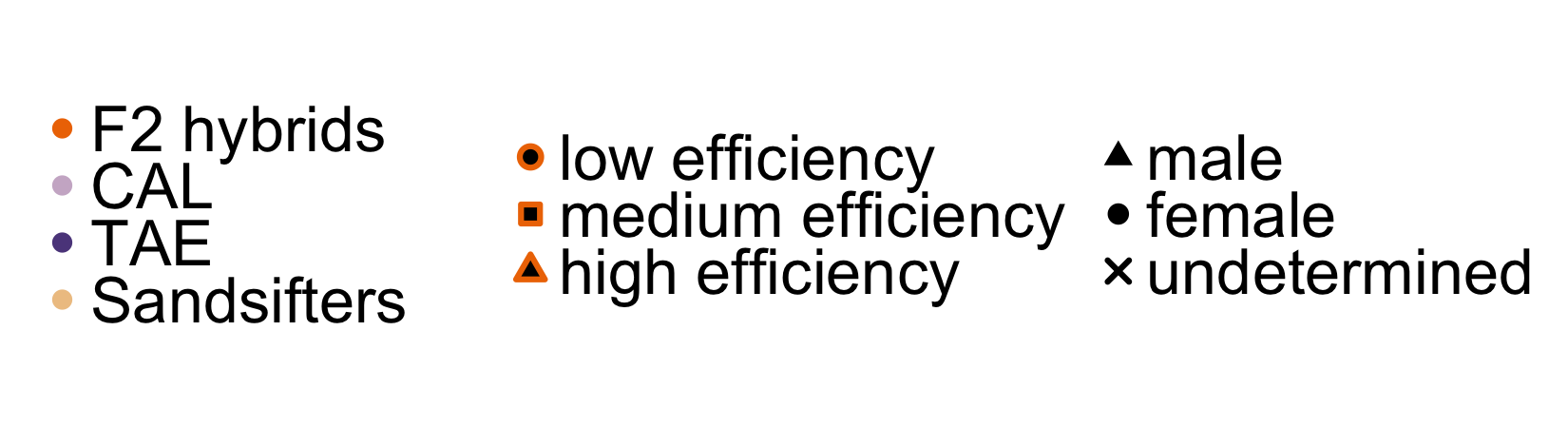


**(b)**

**(a)**

**(c)**

**Figure S3** PCA of shape where the morphospace is defined only by the F2 hybrids (these are the PC scores used for QTL mapping) and the parental individuals are projected onto this space using the predict function. Ellipses represent 95% confidence intervals, and warpgrids show shape differences between mean shape and the most extreme shape at a given PC axes. **(a)** PC1 vs PC2 **(b)** PC3 vs PC4 **(c)** PC 5 vs PC6.
